# Supplementary material for: A large size-selective DNA nanopore with sensing applications
Source: Nat Commun. 2019 Dec 11;10:5655. doi: 10.1038/s41467-019-13284-1 (PMC6906287; doi:10.1038/s41467-019-13284-1)
Supplement: Supplementary file 2 — Supplementary Information [file 41467_2019_13284_MOESM2_ESM.pdf]

Supplementary information

A Large size-selective DNA nanopore with sensing applications

R.P. Thomsen *et al.*

Table of contents

Supplementary Note 1 – Analysis of structural design. **3**
Supplementary Figure 1 – Design optimization of DNA nanopore staples. **3**
Supplementary Note 2 – Free energy of insertion. **4**
Supplementary Figure 2 – Energy considerations. **4**
Supplementary Figure 3 – Hydrophobic modification of flap-channel compartments. **6**
Supplementary Figure 4 – Functionalization of lipid anchors to DNA. **7**
Supplementary Figure 5 – Studies of the effect of increasing number of hydrophobic moieties. **8**
Supplementary Figure 6 – nsTEM gallery of closed DNA nanopore. **9**
Supplementary Figure 7 – nsTEM gallery of opened DNA nanopore. **10**
Supplementary Figure 8 – nsTEM gallery closed lipidated DNA nanopore. **11**
Supplementary Figure 9 – nsTEM gallery of opened lipidated DNA nanopore. **12**
Supplementary Figure 10 – nsTEM gallery of lipidated DNA nanopores incubated with SUVs. **13**
Supplementary Figure 11 – DLS measurements of SUVs created by extrusion using 100 nm, 200 nm and 400 nm filters. **14**
Supplementary Figure 12 – CryoEM images of the DNA nanopore. **15**
Supplementary Figure 13 – Structural stability of DNA nanopore at increasing temperature. **16**
Supplementary Figure 14 – DNA nanopore dimerization kinetics. **17**
Supplementary Figure 15 – FRET studies of DNA nanopore flaps. **18**
Supplementary Figure 16 – Plugging mechanism of DNA nanopores. **19**
Supplementary Figure 17 – Plugging and unplugging of DNA nanopores. **20**
Supplementary Figure 18 – Overview of TIRF setup. **22**
Supplementary Figure 19 – Single particle tracking and drift correction. **23**
Supplementary Figure 20 – Control showing no significant photobleaching of ATTO 655 in the experimental time frame **24**
Supplementary Figure 21 – Study of nanopore docking, insertion and size dependence. **25**
Supplementary Table 1 – Statistics on size distributions for both Alpha-Hemolysin and DNA Nanopore experiments. **26**
Supplementary Figure 22 – Reorientation and pore formation kinetics upon binding to SUVs. **27**
Supplementary Figure 23 – TIRF key experiments. **28**
Supplementary Figure 24 – Stability of DNA nanopore in TIRF solution. **29**
Supplementary Figure 25 – Confocal setup. **30**
Supplementary Figure 26 – Non-filled and leaky CLSM traces of SRB and 40kDa dextran-FITC inside GUVs. **31**
Supplementary Table 2 – CLSM normalized intensities of internal color (dFITC-40k) **32**
Supplementary Table 3 – CLSM normalized intensities of internal color (dFITC-500k) **33**
Supplementary Table 4 – CLSM normalized intensities of internal color (dFITC-40k + plug) **34**
Supplementary Figure 27 – Real-time sensing of "unplugging" oligonucleotide. **35**
Supplementary Table 5 – Registered dye flow events in real-time sensing assay sorted by event type. **36**
Supplementary Figure 28 – Control for real-time sensing with non-plugged DNA nanopores. **37**
Supplementary Figure 29 – Control for real-time efflux of ATTO 655 via the α-hemolysin pore. **38**
Supplementary Figure 30 – Loading efficiency of various dye encapsulated SUVs. **39**
Supplementary Note 3 – Numerical relation between observed flow rate and the pore size described by the Hagen-Poiseuille relation. **40**
Supplementary Note 4 – Freely diffusion model of the translocated dye molecules. **41**
Supplementary Note 5 – Synthesis of Azido-palmitoyl building block ((S)-1-azido-3-(palmitoyloxy)propan-2-yl sulfate). **43**
Supplementary Figure 31 – CaDNAno design of DNA nanopore. **45**
Supplementary Note 6 – DNA origami staples. **46**
Supplementary Table 6 – DNA sequences. **46**
Supplementary References **50**

Supplementary Note 1 – Analysis of structural design.

As several features was desired for the design of the nanopore, including flaps, inner channel functionality and crossovers, it was important for us to ensure that the designed staples were incorporated according to known important parameters to avoid apparent kinetic and thermodynamic folding traps. As a result, R.S.S. wrote a CaDNAno[1] plugin termed “Staplestatter” available as GitHub repository (https://github.com/scholer/staplestatter). Usage allow fast overview of staple patterns and stretches which further can be used to get an overview of melting temperatures etc.

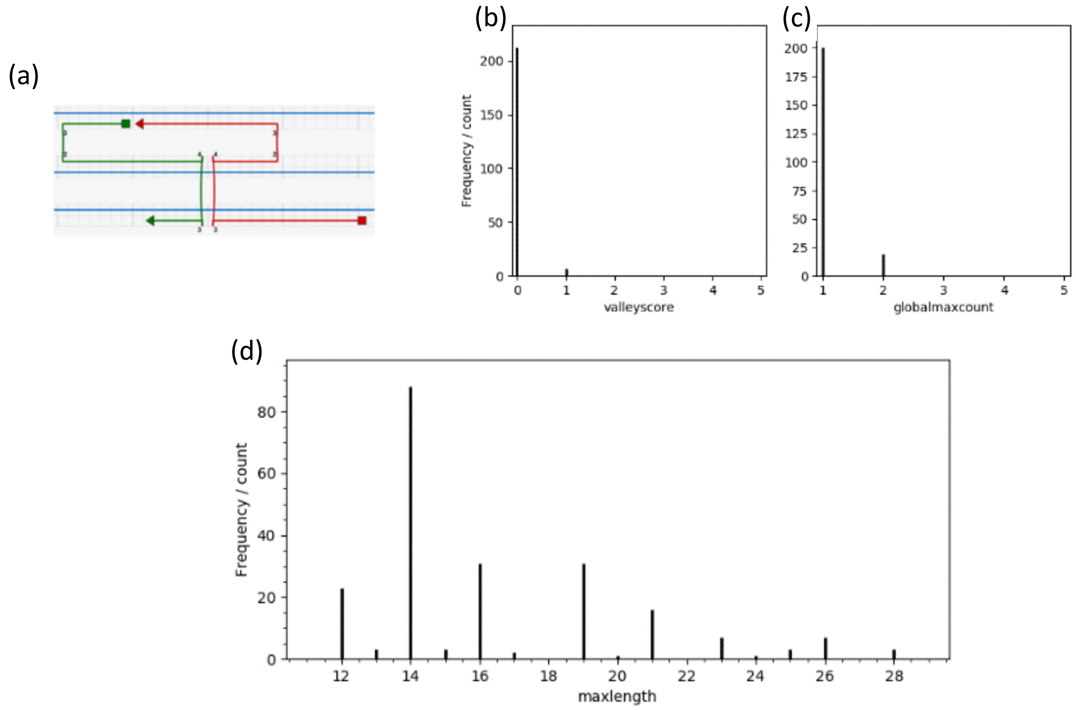

Supplementary Figure 1 – Design optimization of DNA nanopore staples.

(a) Example of 2 strands where the green strand has a desirable long nucleation site of 16-nts with two shorter 8-nt sequences flanking. Meanwhile, the red staple contains an undesired structural “valley” - a short segment flanked by two long stretches. (b) The number of strands with “valley” was kept to a minimum and analyzed by a CaDNAno plugin written by R.S.S. The lower the score the better to avoid kinetic folding traps. (c) Analysis of the number of global max lengths of the staples. Optimally a single site is desired since it indicates a single “nucleation” site. (d) Analysis of the longest continues segment of individual staple strands to ensure all strands have sufficiently long nucleation site. With the plugin the T_M of segments can also be calculated by input of the scaffold sequence.

Supplementary Note 2 – Free energy of insertion.

To overcome the penalty of forming a pore in a lipid bilayer a compensatory energetic gain is required. Using DNA nanopores modified by lipids have demonstrated to be a feasible approach as they provide their solvation energies provide the required energetic gain if adapted to size. In this paper we use lipid moieties of cholesterol and palmitoyl. In previous papers this consideration has been addressed using continuum models[2] or coarse grained molecular dynamic simulations[3].

The energy required in the formation of a cylindrical membrane pore is dependent on the pore radius (R) and the bilayer tensions (γ). Using continuum models[4], the energy requirement can be assumed to be linearly dependent on the pore size and the line tension (γ_L) which describes the cost of the exposed bilayer circumference. Importantly, the exact γ_L values are tied to the specific lipid composition and pore structure, with typical values in the range of 5–40 pN[4]. The surface tension (γ_S) on the other hand describe the gain from minimizing the membrane area however this can be neglected for small pores due to the radius squared. Using the continuum model, we get a linear relationship between required energy and pore radius at the nanoscale:

$$E(R) = (2\pi\gamma_L R) - (\pi\gamma_S R^2) \approx (2\pi\gamma_L R), \text{ for R in nm range}^4 \qquad (1)$$

Similarly, coarse grained molecular dynamic simulations have previously also provided a linear relationship between pore radius and the energy requirement[3].

As the solvation energy of lipids can be determined, the amount of hydrophobic tags needed can be estimated from equation 4.2, or from coarse grained molecular dynamics simulations. While we have not been able to find any good measurements of the solvation energy regarding palmitoyl, cholesterol contributes with a free energy gain of up to -75 kJ/mol upon bilayer insertion[3]. Based on the assumption of a similar gain for each palmitoyl, an estimated 2.5-3 moieties are necessary each radial nm increment. While we have not done a systematic study to establish the best mixture between palmitoyl and cholesterol for most optimal insertion, we experience the most efficient insertion with all lipids together.

The plot of previous and our DNA origami pores based on the continuum model and coarse grained molecular dynamic simulations have been plotted in Supplementary Figure 2. They show how all DNA origami pores reported are positioned in the energetically favorable region. Importantly these considerations only focus on the stability of the final state of a formed cylindrical pore and does not consider initial nucleation barriers or other related penalties.

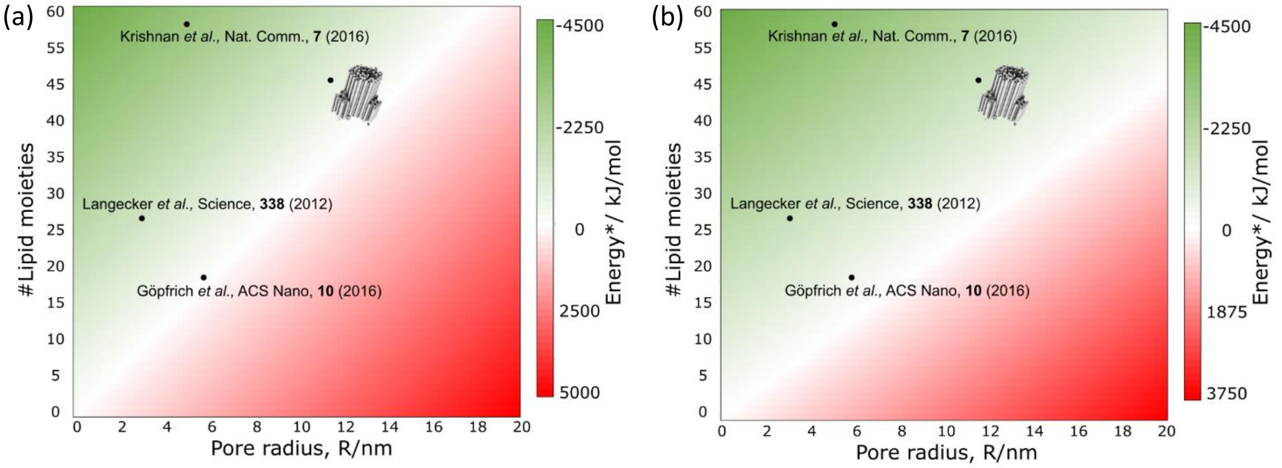

Supplementary Figure 2 – Energy considerations.

Plot of free energy landscape between pore size and the number of lipid moieties based on a continuum model $E(R) = (2\pi\gamma_L R) - (\pi\gamma_S R^2) \approx (2\pi\gamma_L R)$, for R in nm range. (a) or molecular dynamics simulations (b). (a) Continuum model considerations has been described before by Langecker *et al.*[2] The plot has adapted a moderate

γ_L-value of 22.5 pN. (b) The MD simulations is adapted from work by Göpfrich *et al*.[3]. While the DNA nanopores plotted does not use the same lipidation moieties, cholesterol is used as model to estimate the free energy gain per lipid moiety, with a free energy of solvation into bilayers of *E(n) = -75k J/mol. Three additional nanopores have been included to compare the theoretical considaretions[2,3,5]. Theoretically, the 46 hydrophobic moieties are more than enough to stabilize the large 22 nm bilayer pore.

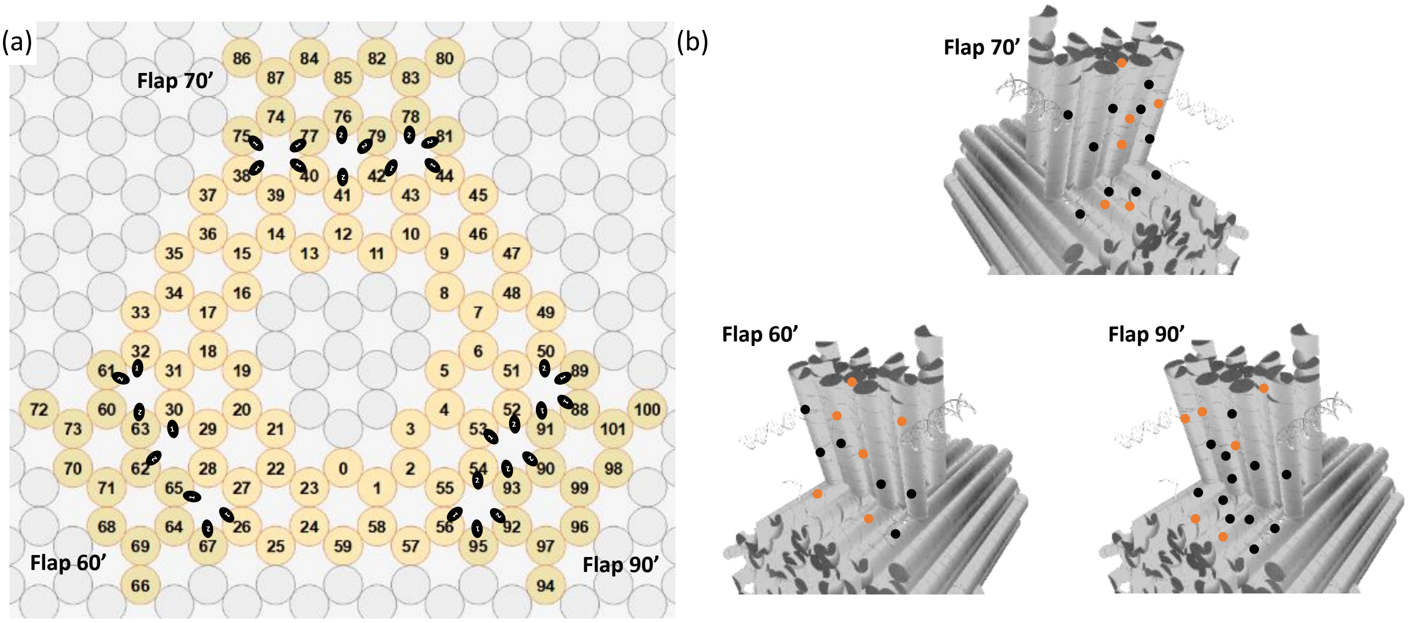

Supplementary Figure 3 – Hydrophobic modification of flap-channel compartments.

(a) Top view of all positioned lipid moieties in the flap-channel system. Some helices have 1 while others have 2 hydrophobic moieties. 17 are on the channel while 29 is on the flaps. 6 of the modifications on each flap are cholesterol (bought from IDT as 3’-cholesterol TEG) while the 28 remaining are modified with a palmitoyl anchor using click chemistry. See Supplementary Figure 4 and materials and methods. (b) View of modification of each flap-channel system. Positions of cholesterol modifications are colored orange, while palmitoyl is black.

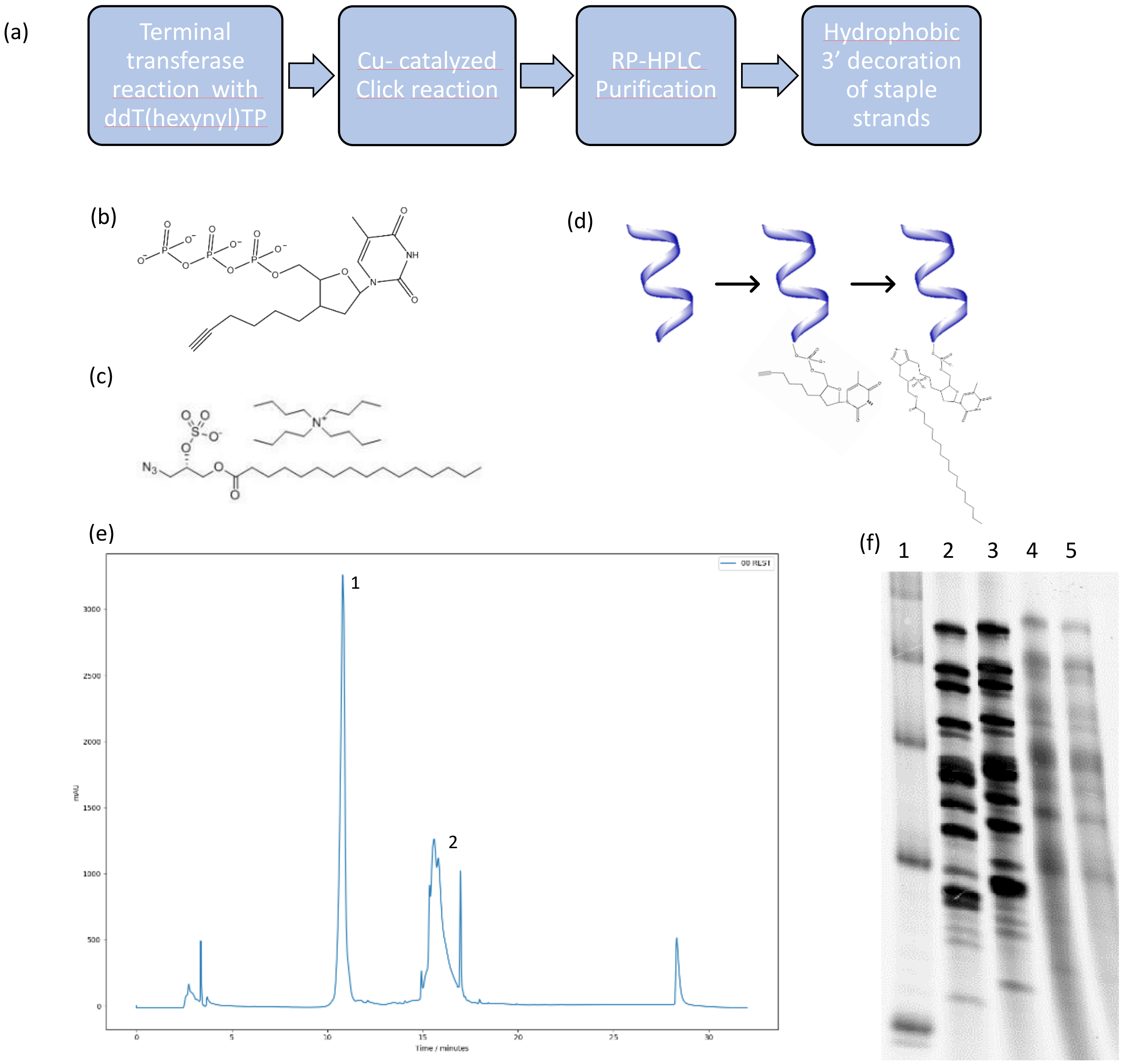

Supplementary Figure 4 – Functionalization of lipid anchors to DNA.

(a) Synthesis strategy of lipidated oligonucleotides. (b) The ddT(hexynyl)TP substrate to be attached with terminal transferase (TdT). (c) Palmitoyl lipid anchor attached by Cu-catalyzed conjugation to the alkyne of ddT(hexynyl)TP at the 3’ end of the select staples. (d) Reaction flow of DNA strands, in a one-pot reaction – pulled staple strands. (e) RP-HPLC chromatogram of lipidated strands. Peak 1 are the unmodified plain oligonucleotides eluted after about 11 minutes. The broad peak 2 are the collected fraction of lipidated oligos used for functionalization. (f) 16% Denaturing gel of 28 strands which is modified with the palmitoyl moieties attached. Lane1: 10bp ladder. Lane2: 28 plain oligonucleotides. Lane3: Terminal transferase reacted oligonucleotides. Lane4: Cu-click reacted strands from 3. Lane5: RP-HPLC purified strands. Length of the 28 oligos before TdT reaction: 56-nts: 2 strands, 50-nts: 1, 49-nts: 1, 48-nts: 1, 47-nts: 1, 46-nts: 2, 42-nts: 2, 39-nts: 1, 38-nts: 3, 37-nts: 1, 35-nts: 2, 33-nts: 2, 30-nts: 1, 28-nts: 3, 27-nts: 1, 26-nts: 2, 25-nts: 1 and 22-nts: 1.

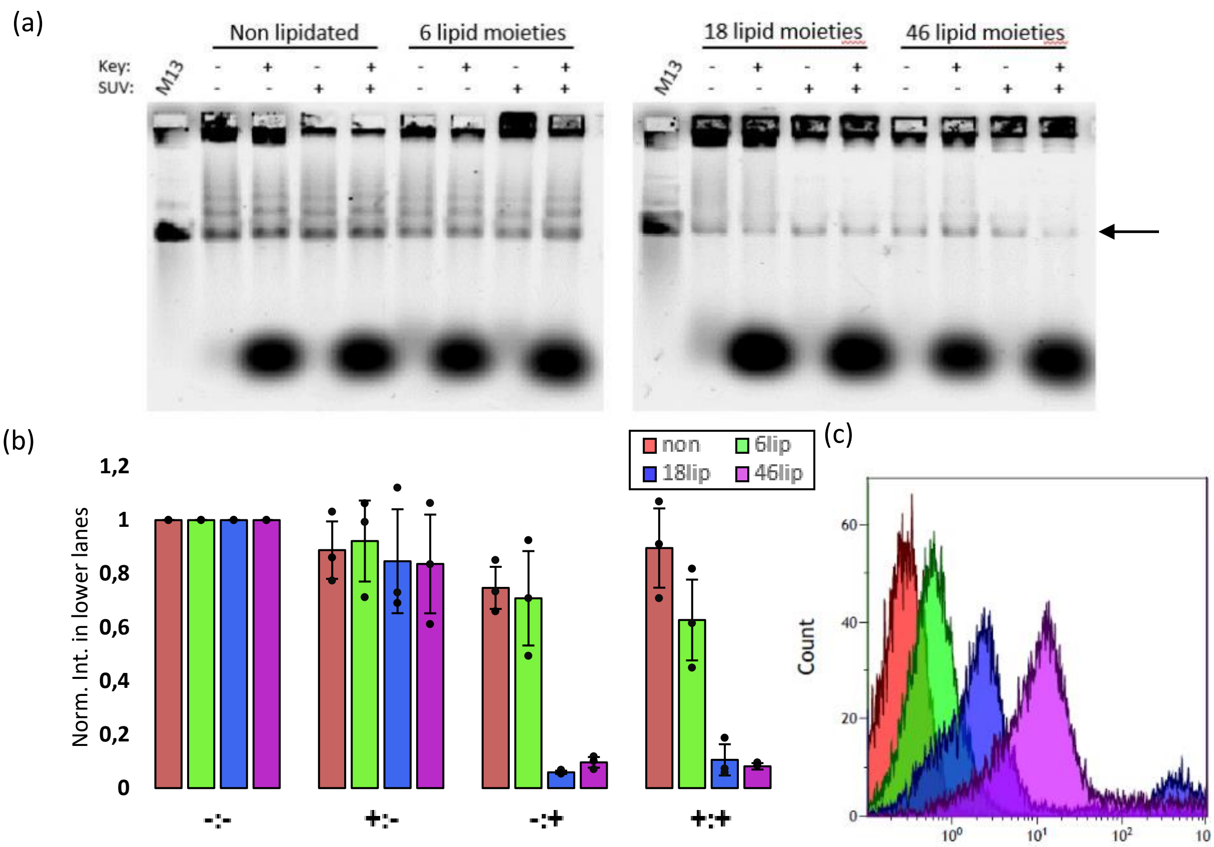

Supplementary Figure 5 – Studies of the effect of increasing number of hydrophobic moieties.

(a) 1% AGE of ATTO 488 labelled DNA nanopores with 0, 6, 18, or 46 lipid moieties attached. Legend denotes whether opening key has been added (+) or not (-); and whether the structures have been incubated with 200 nm extruded POPC SUVs for 1,5 h (+) or not. Arrow indicates monomer structure of interest. (b) ATTO 488 quantification of monomer band in AGE (arrow) which are normalized to each (-:-) control lane. Legend: **Key addition : SUV addition**. Independent replicates of 3 gels scanned with a typhoon scanner and quantified by ImageQuant software (GE healthcare) Error bars indicate standard deviation. (c) Flow cytometry of GUVs created by the inverted emulsion method[6] (also used for CLSM), incubated with opened ATTO 488 labelled DNA nanopores containing, 0 (red), 6 (green), 18 (blue) and 46 (magenta) lipid moieties.

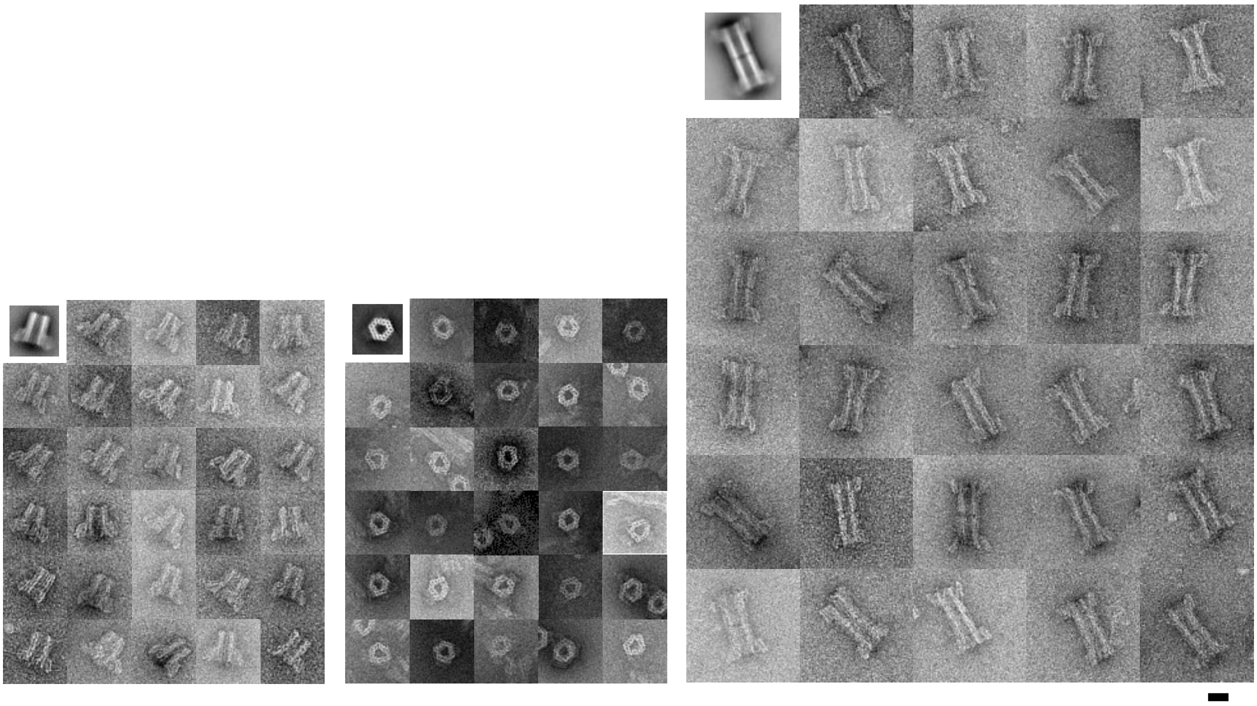

Supplementary Figure 6 – nsTEM gallery of closed DNA nanopore.

Gallery of DNA nanopore in the closed flap state. Monomers observed from side-view (a), and top-view (b) along with 2D class average in upper left corner. (c) Dimerized closed DNA nanopores observed from side-view along with 2D class average in upper left corner. Scale bar is 20 nm.

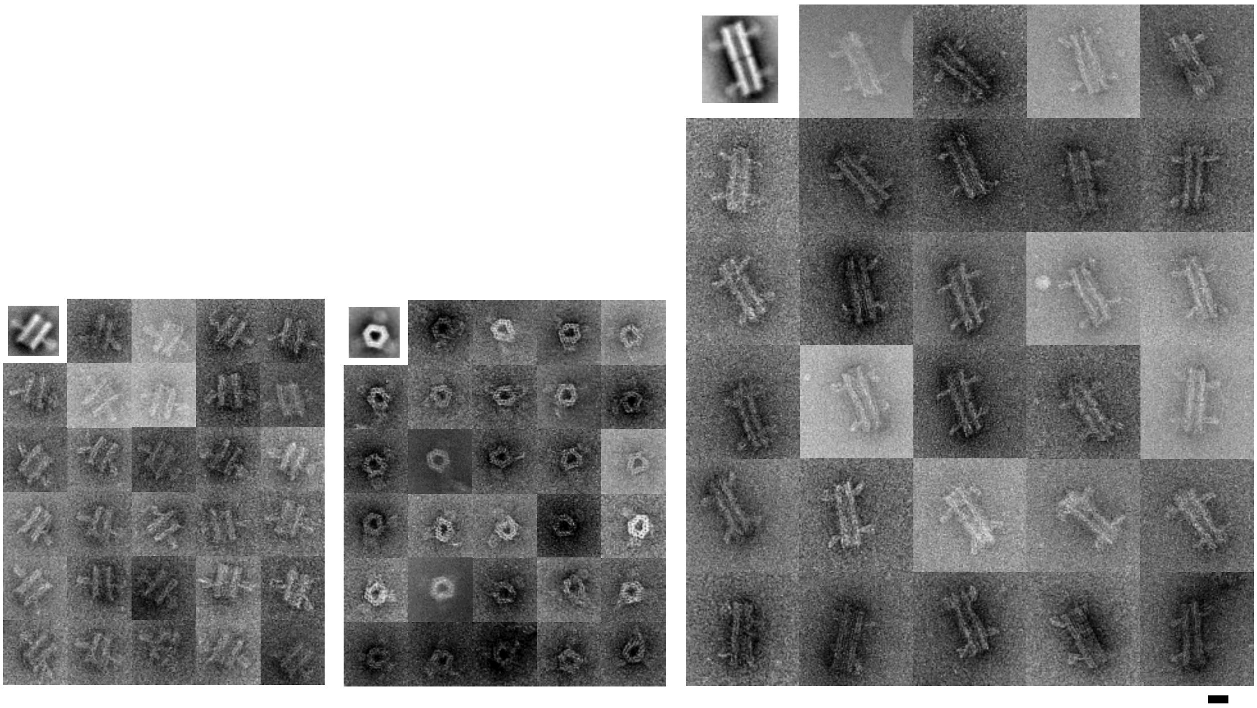

Supplementary Figure 7 – nsTEM gallery of opened DNA nanopore.

Gallery of DNA nanopore in the opened flap state. Monomers observed from side-view (a), and top-view (b) along with 2D class average in upper left corner. (c) Dimerized opened DNA nanopores observed from side-view along with 2D class average in upper left corner. Scale bar is 20 nm.

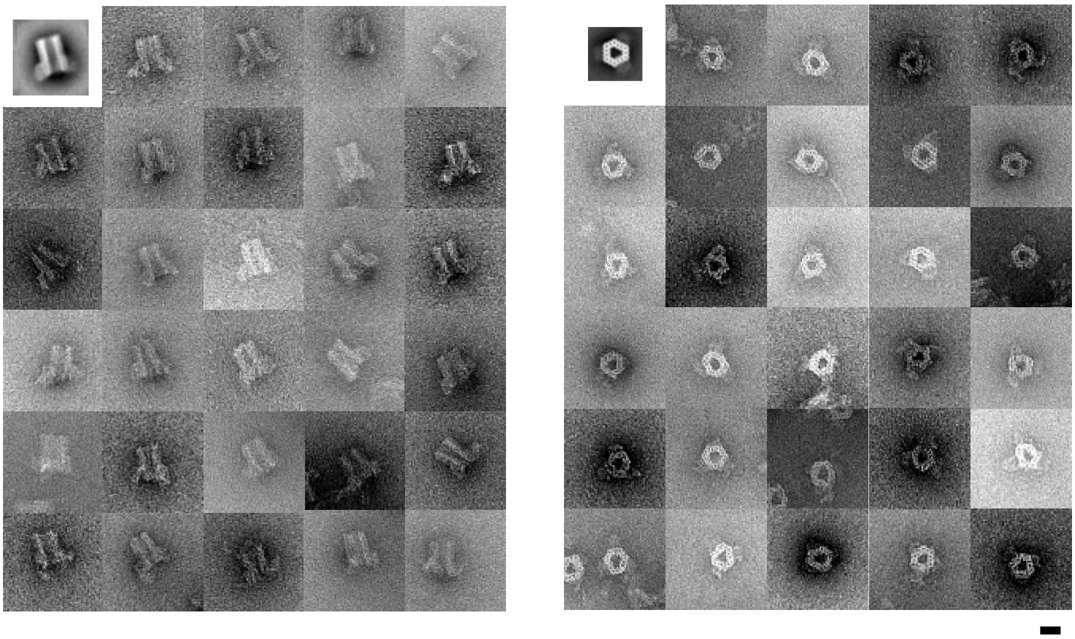

Supplementary Figure 8 – nsTEM gallery closed lipidated DNA nanopore.

Gallery of lipidated DNA nanopore in the closed flap state. Monomers observed from side-view (a), and top-view (b) along with 2D class average in upper left corner. Scale bar is 20 nm.

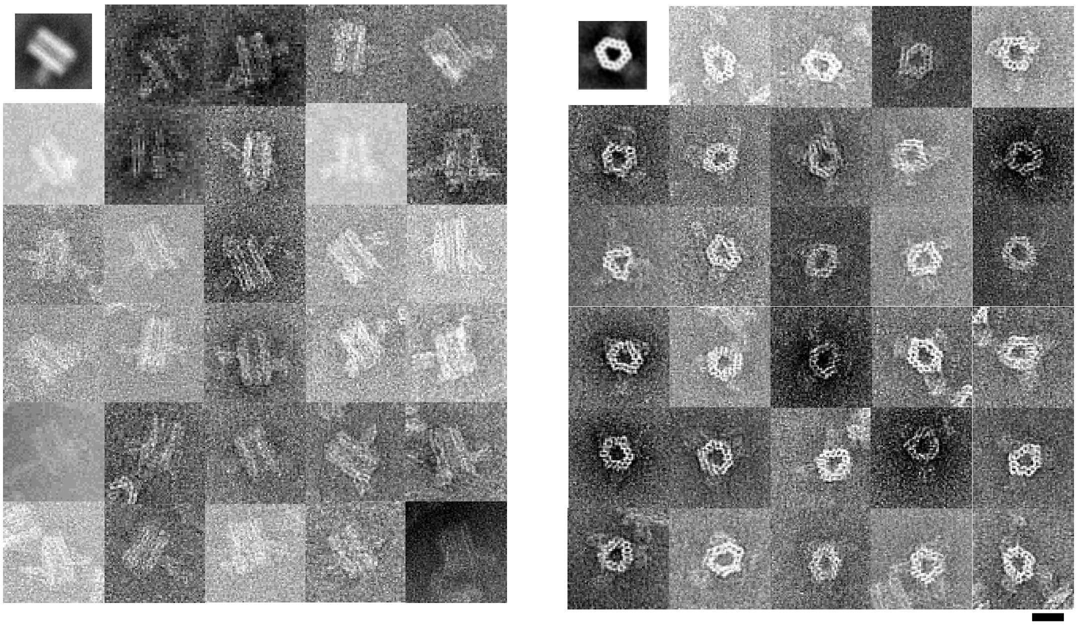

Supplementary Figure 9 – nsTEM gallery of opened lipidated DNA nanopore.

Gallery of lipidated DNA nanopore in the opened flap state. Monomers observed from side-view (a), and top-view (b) along with 2D class average in upper left corner. Scale bar is 20 nm.

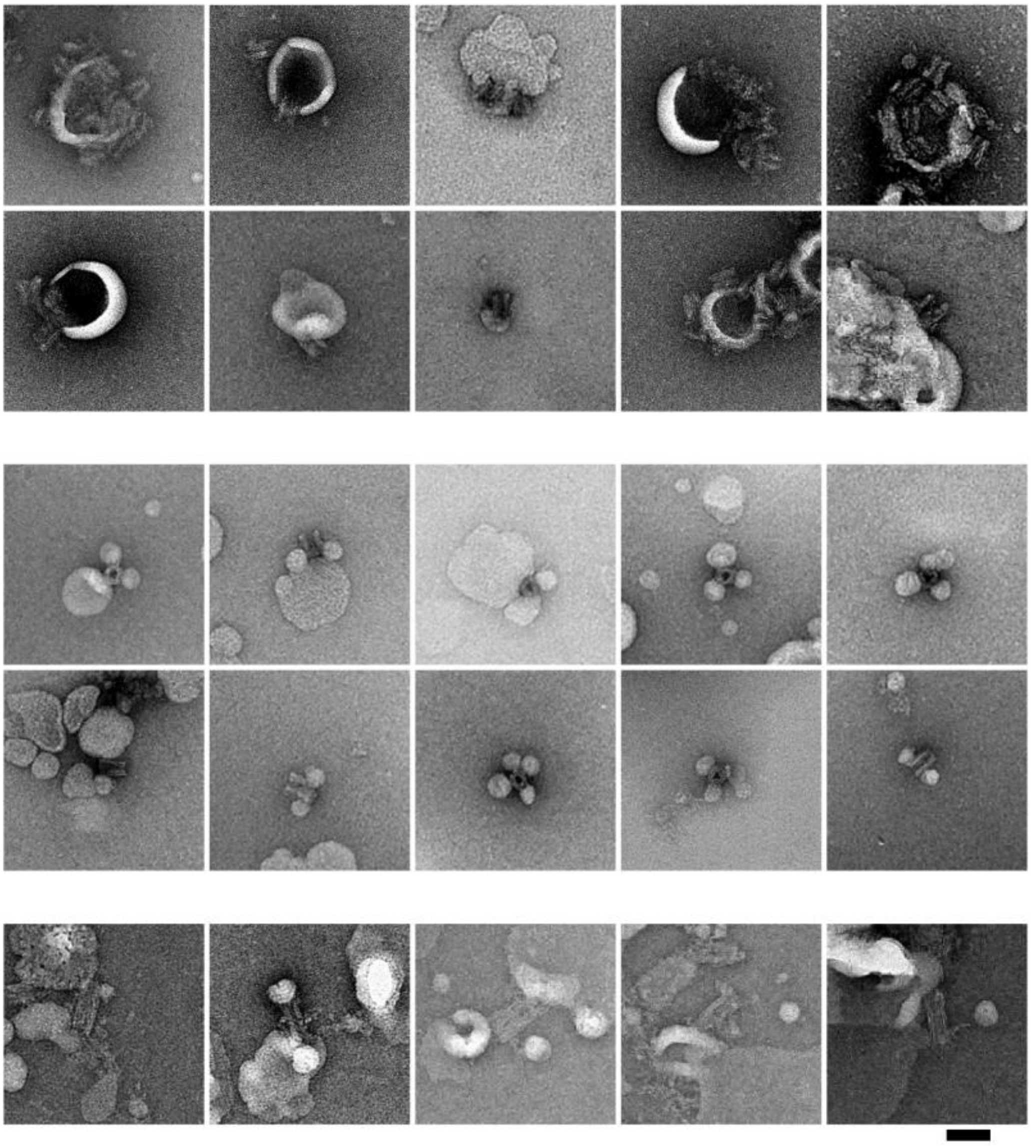

Supplementary Figure 10 – nsTEM gallery of lipidated DNA nanopores incubated with SUVs.

Upper panel: Inserted DNA nanopores into SUVs.

Mid panel: DNA nanopore flaps interacting with SUVs without insertion.

Lower panel: Dimerized DNA nanopores inserted into liposomes.

In general, smaller SUVs seems to be able to passivate insertion by interacting favorably with hydrophobic flap-channel compartments. Meanwhile, bigger SUVs are penetrated to a much larger extend. Scale bar is 50 nm.

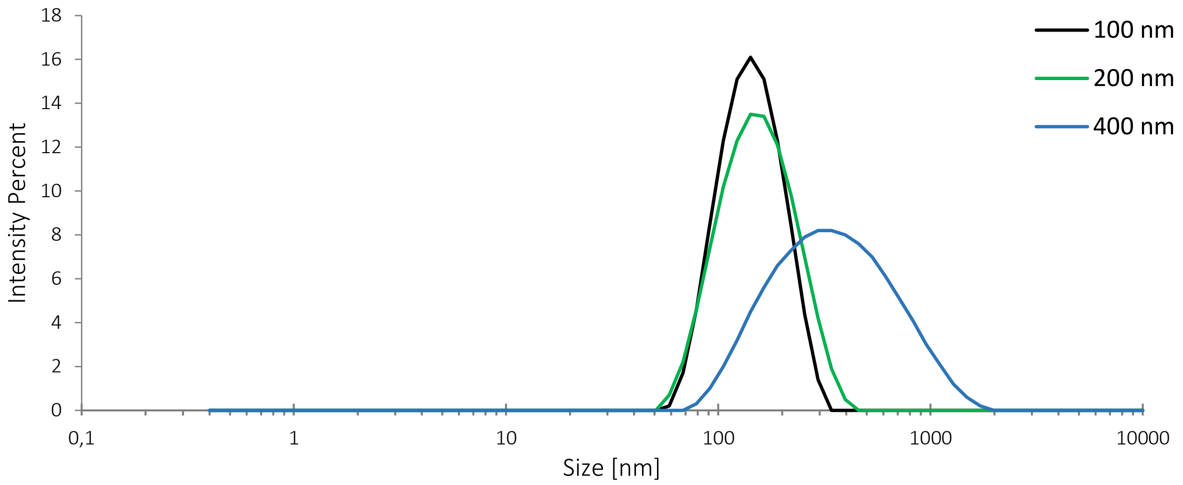

PC membrane size	100 nm ◆	200 nm ◆	400 nm ◆
**Avg. Peak size**	148.8 nm	163.0 nm	417.2 nm
**PDI**	0.094	0.134	0.269

Supplementary Figure 11 – DLS measurements of SUVs created by extrusion using 100 nm, 200 nm and 400 nm filters.

Extrusion was done with up to 1 ml of 2 mg/ml POPC emulsion sample in the assembled Avanti aperture with PC membrane filters containing 100-400 nm sized pores depending on desired diameter of the liposomes. Liposome samples were then passed back and forth a total of 13 times and size was checked by DLS analysis. For the Analysis the sample was diluted to 0.2 mg/ml.

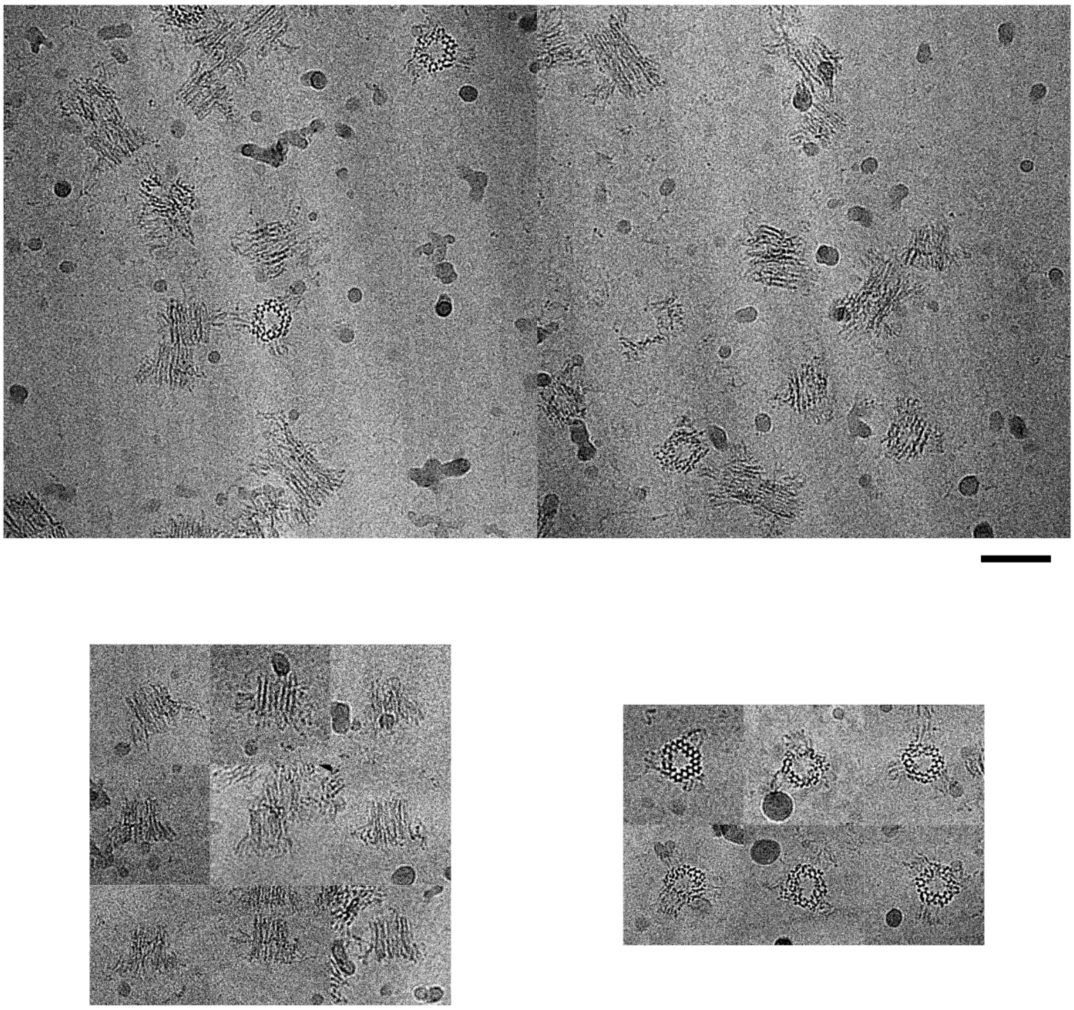

Avgerage Length	n
31.6 nm ±1.4 nm	45

Supplementary Figure 12 – CryoEM images of the DNA nanopore.

Two Cryo EM images of the DNA nanopore on prepared quantifoil grids. Below are cutouts of DNA nanopore monomers from side- and top-view. While the 2D-class average side-view showed a length of 35 nm, quantification of 45 individual DNA nanopore monomers yielded an average length of 31.61 nm ±1.38 nm was measured, which is very close to the expected 32 nm. Scale bar is 50 nm.

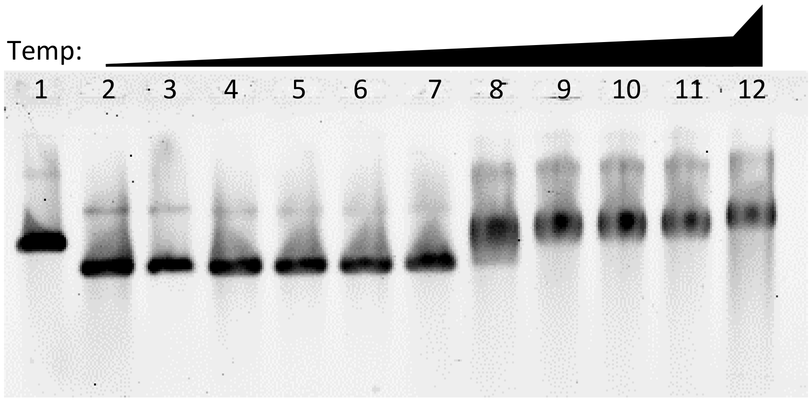

Supplementary Figure 13 – Structural stability of DNA nanopore at increasing temperature.

1% Agarose gel electrophoresis of DNA nanopores incubated at the increasing temperature for 30 minutes before running the gel. (1) M13, (2-12) DNA nanopore at RT (2), 40 °C (3), 50 °C (4), 52 °C (5), 54 °C (6), 56 °C (7), 58 °C (8), 60 °C (9), 62 °C (10), 64 °C (11), 90 °C (12).

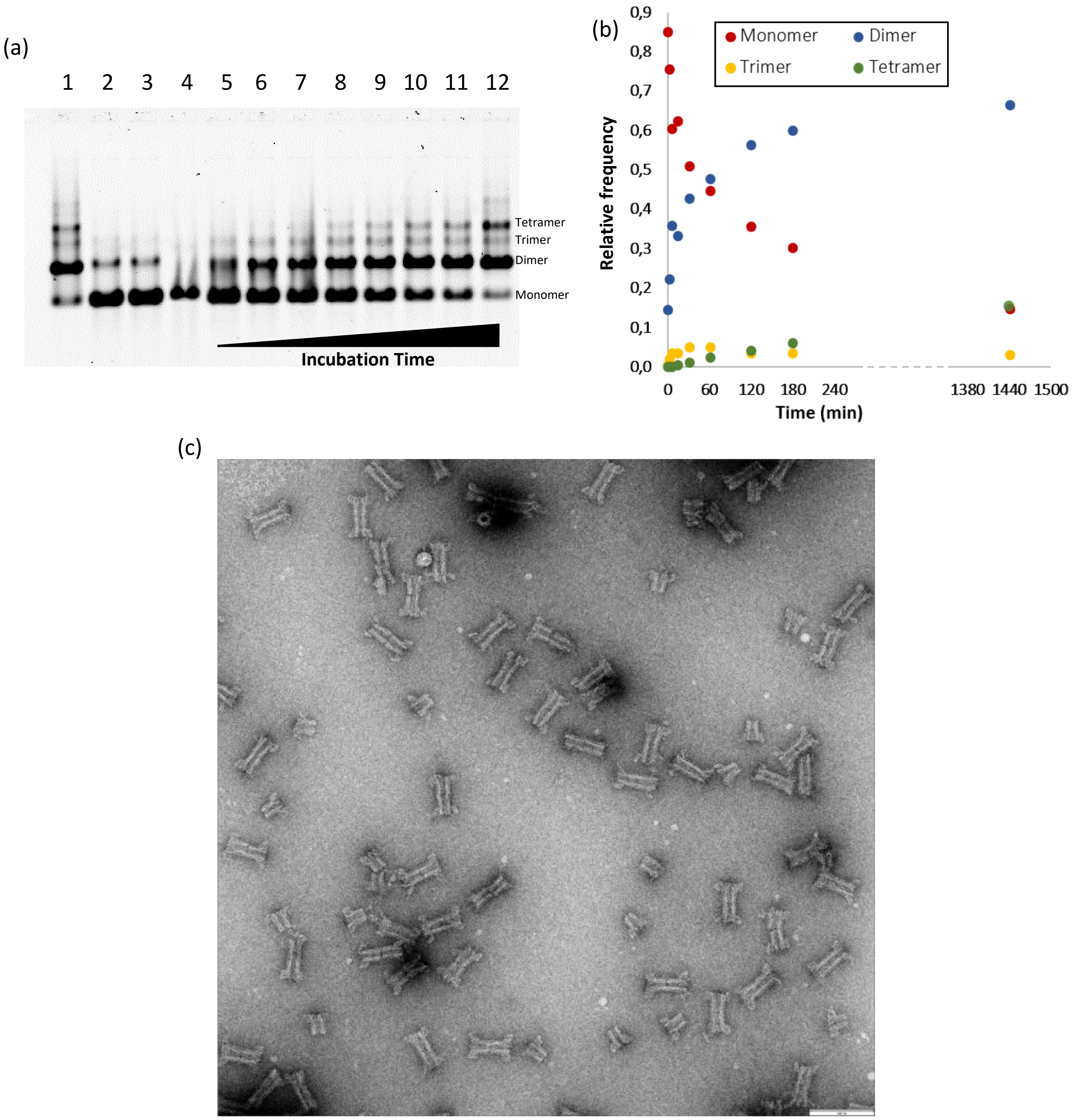

Supplementary Figure 14 – DNA nanopore dimerization kinetics.

(a) 1% AGE of A and B versions of the DNA nanopore. Lane2 and 3 is A and B monomers alone respectively, incubated for 30 min. Lane 4: M13, Lane 5-12 and lane 1 are A and B incubated for 0min, 5min, 15min, 30min, 60min, 120min, 180min, 24h at 40C and 6days at RT. (b) Quantification of monomers, dimers, trimers and tetramer bands from the Agarose gel. A plateau is reached at around 70%. From the gel unspecific aggregation is apparent but sticky-end dimerization has a significant effect. (c) Raw nsTEM image of nanopore incubated with matching sticky ends scale bar is 100 nm.

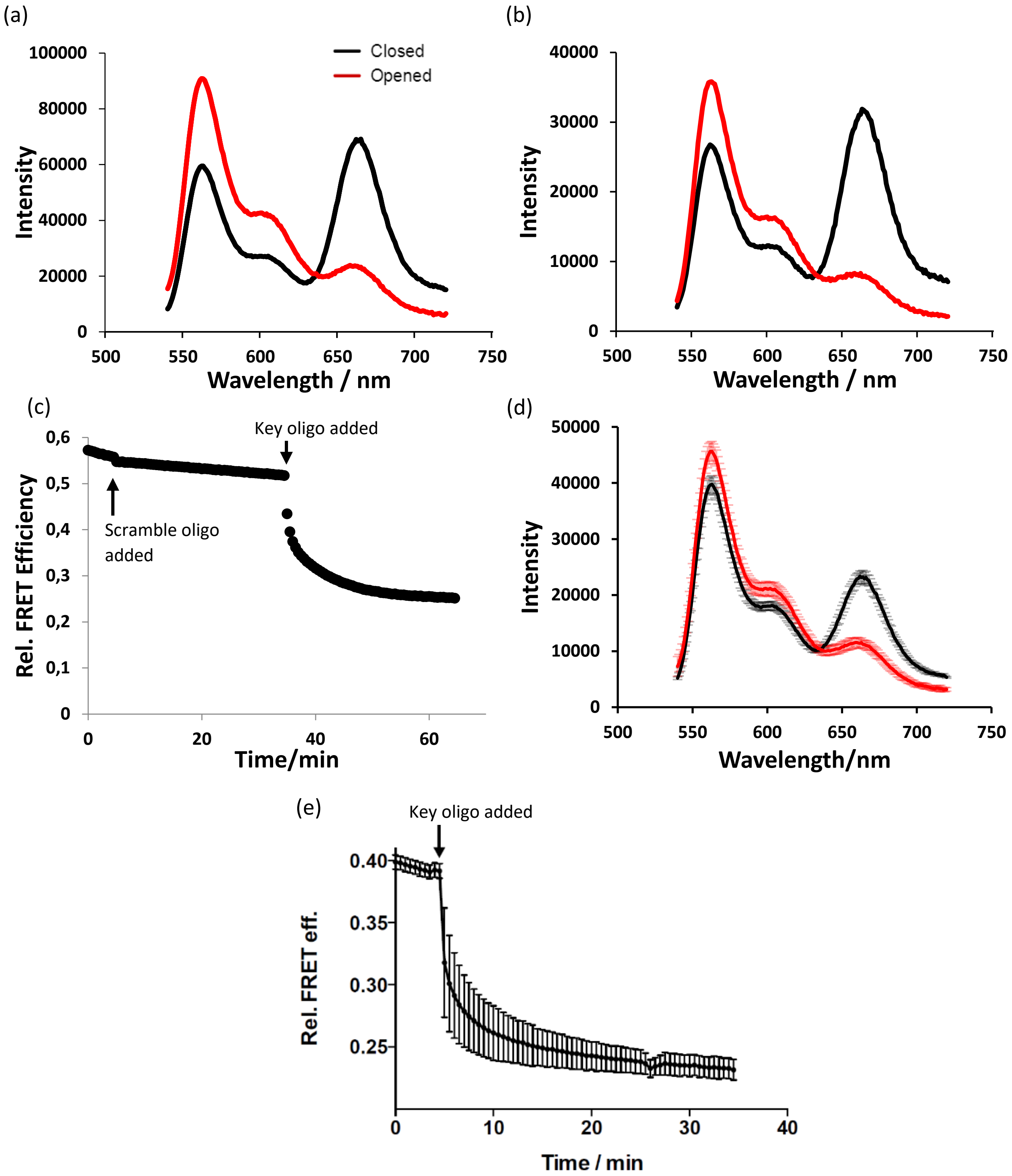

Supplementary Figure 15 – FRET studies of DNA nanopore flaps.

(a) FRET of DNA nanopore with fluorophore in flap-channel system annealed in purified in $TAEM_5N_5$. (b) FRET of DNA nanopore with fluorophore in flap-channel system annealed in purified in KCl. (c) Specificity and kinetics of lock. (d) FRET of lipidated DNA nanopore similar setup to (a). (e) Kinetics of flap opening for lipidated DNA nanopore. (d) and (e) are averages of 3 separate measurements with the standard deviations as error bars. Staple strands used in the FRET measurements are listed in Supplementary Table 3.

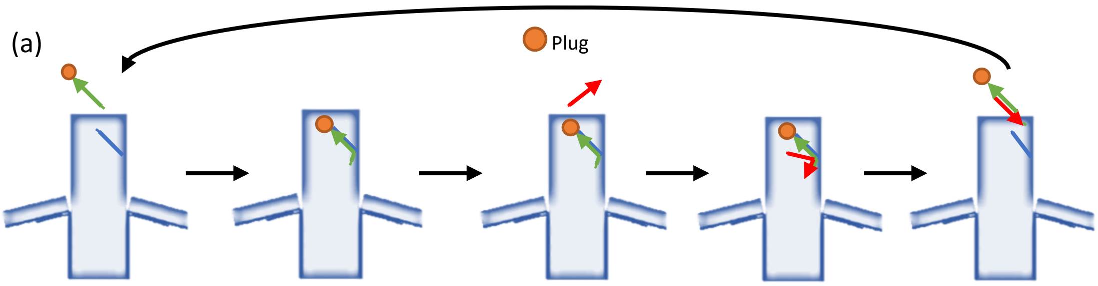

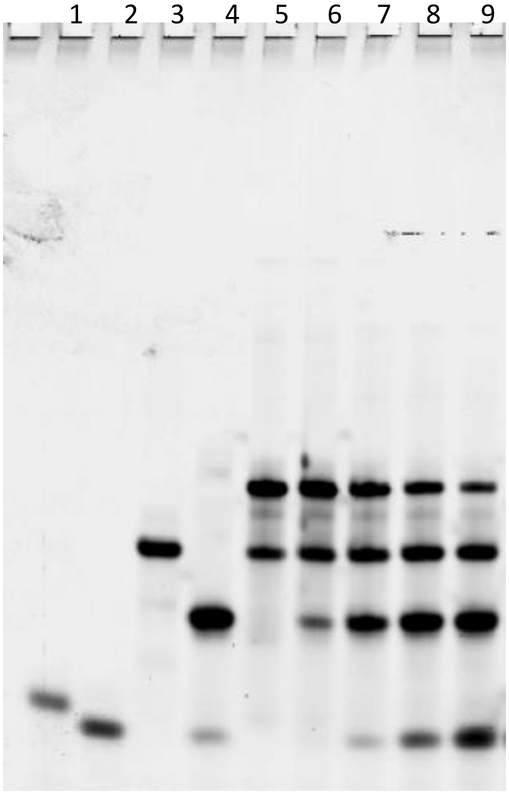

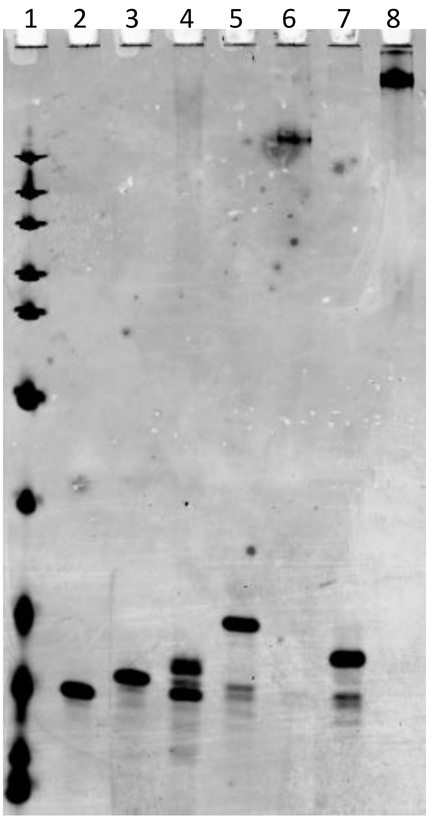

Supplementary Figure 16 – Plugging mechanism of DNA nanopores.

(a) Mechanism of plug capture and unzipping of DNA nanopore. (b) 10 % native PAGE of the plug and unzip mechanism. Lane 1: Plug oligo (green). Lane 2: unplugging oligo (red). Lane 3: Inner channel capture oligo (blue). Lane 4: Plug and unplugging oligos. Lane 5: Plug and inner capture oligos. Lane 6-9: Plug, unplug and capture oligos incubated with increasing unplug oligo excess of 0.1x, 0.5x, 1x or 2x, respectively. Mixtures of oligos in lanes 4 and 5 have been incubated 20 minutes. Mixtures of oligos in lanes 6-9 have been premixed 20 minutes with plug and inner capture strands, followed by 20 min of incubation with unplugging oligos in the given excess. (c) Analysis of modified plug strands using 12% denaturing PAGE. Lane 1: generuler. Lane 2: Plug oligo oligo. Lane 3: 3’ NH_2 plug oligo. Lane 4: NHS-DBCO modified plug oligo. Lane 5: Az-biotin modified oligo. Lane 6. As lane 5 but incubated with streptavidin. Lane 7: Az-lipid modified oligo. Lane 8: Az-PEG (20kDa) modified oligo.

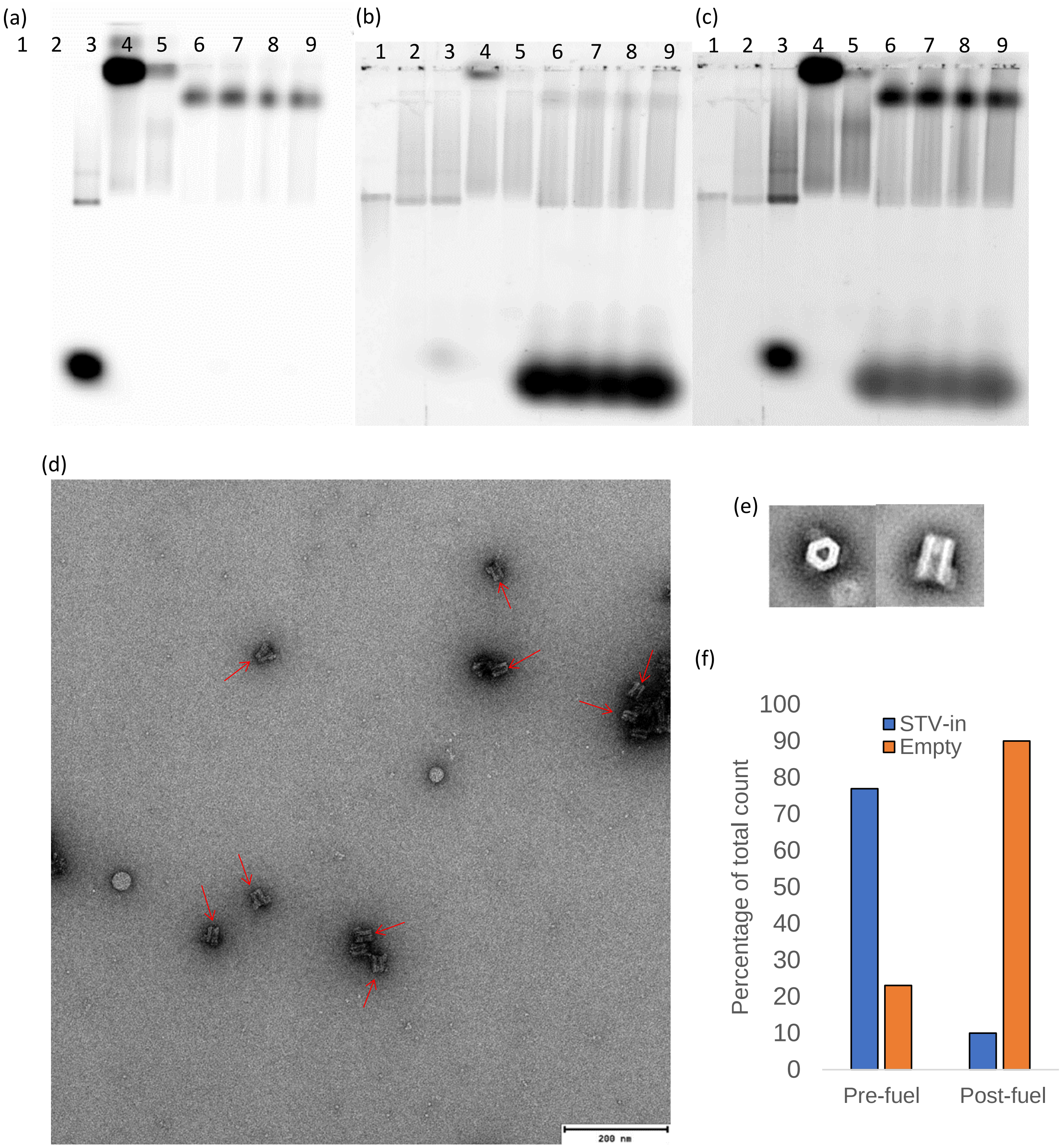

Supplementary Figure 17 – Plugging and unplugging of DNA nanopores.

(a-c) 1% AGE of plugged and unplugged DNA nanopores with 3’-Cy5-Plug scanned for Cy5 before Sybr Gold staining (a) or after Sybr Gold staining for Sybr Gold (b) and 488 nm to Cy5 (c). Lane 1: M13. Lane 2: DNA nanopore without plug. Lane 3: DNA nanopore with 3’-Cy5-DNA plug strands. Lane 4: DNA nanopore with 3’-Cy5-DNA-PEG plug strand. Lane 5: DNA nanopore with 3’-Cy5-DNA-PEG plug strand (+). Lane 6-9: DNA nanopore with 3’-Cy5-DNA-PEG plug strand (+) incubated with the “unplugging” strand for 30, 15, 5, or 1 minute(s), respectively. Samples 5-9 has been purified after plug addition, denoted by (+). The plug is nicely integrated and provides a Cy5 fluorescence of nanopore (3 vs. 4). By using PEG plug strand the migration is becomes slower, while maintaining

Cy5 tagged structures by plug incorporation (4 and 5 vs. 3). Incubation with the “unplugging” strand quickly detaches the nanopore plug and revert the nanopore migration and releases Cy5-tagging. (d) Raw nsTEM image of DNA nanopores with streptavidin immobilized in inner pore for visualization. Arrows indicate immobilized streptavidin in the nanopore channel. Scale bar is 200 nm. (e) Single particle class average of streptavidin-plugged DNA nanopores from top- and side-view. (f) Quantification of efficiency of plugging and unplugging from several TEM images. Biotin oligo - green in (a) incubated for 30min before addition of streptavidin for 1 hour. Incubation with “unplugging” strand - red in (a) - was done for another hour before imaging.

(a)

(b)

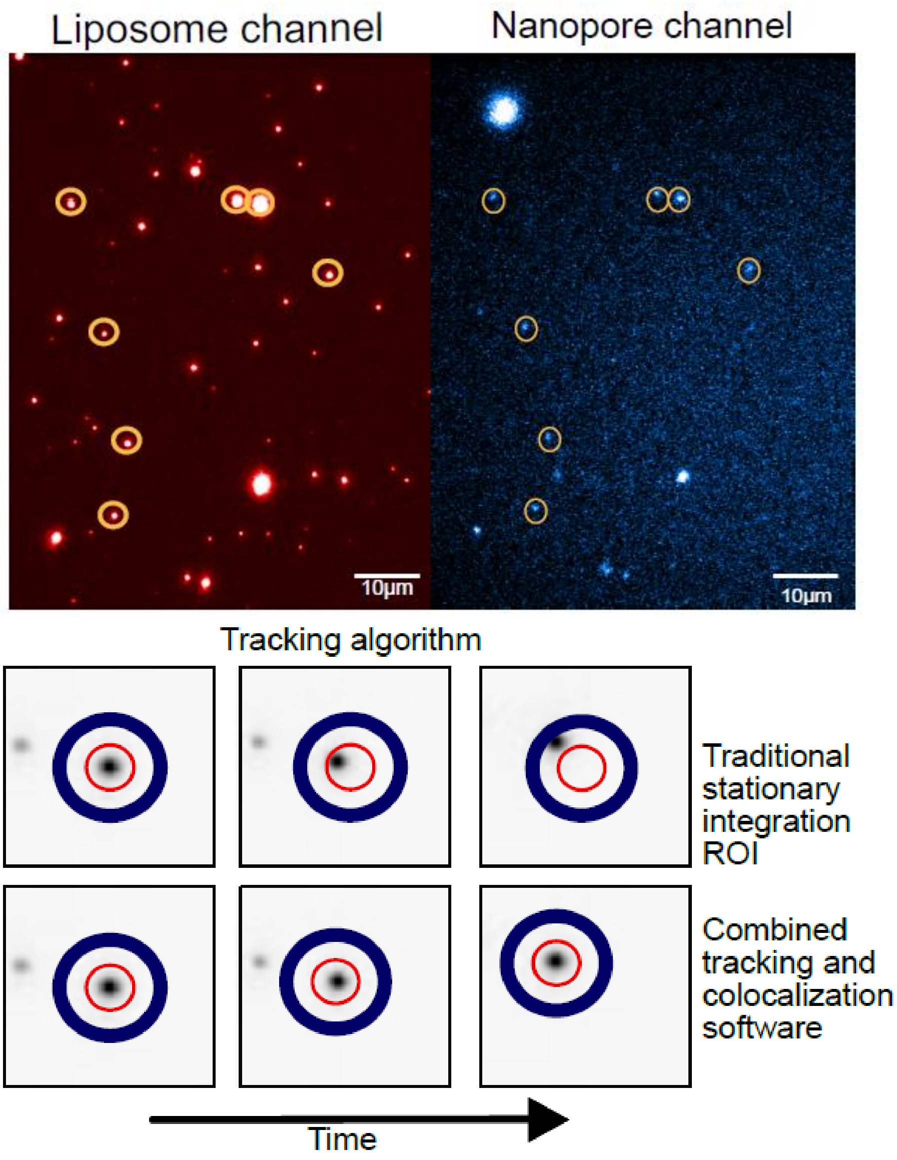

Supplementary Figure 18 – Overview of TIRF setup.

(a) Real image of the liposome channel (left) and the nanopore channel (right) used to correlate the docking of nanopores to specific liposomes, here highlighted in circles. Using simultaneous imaging, it is possible to, through this correlation, directly observe docking and leaking kinetics for individual liposomes. (b) Schematics of tracking algorithm used to stabilize time dependent movements. Due to long experimental timeframes (+9 hours), inevitable drifting in the x-y directions was observed. To correct for this, without biasing data, we deployed single particle tracking (SPT), that allowed us to follow the movement of liposomes throughout the experiment and hence correct for any drift present. Essentially this allows the integration area to move with the liposome, instead of attaining a stationary position.

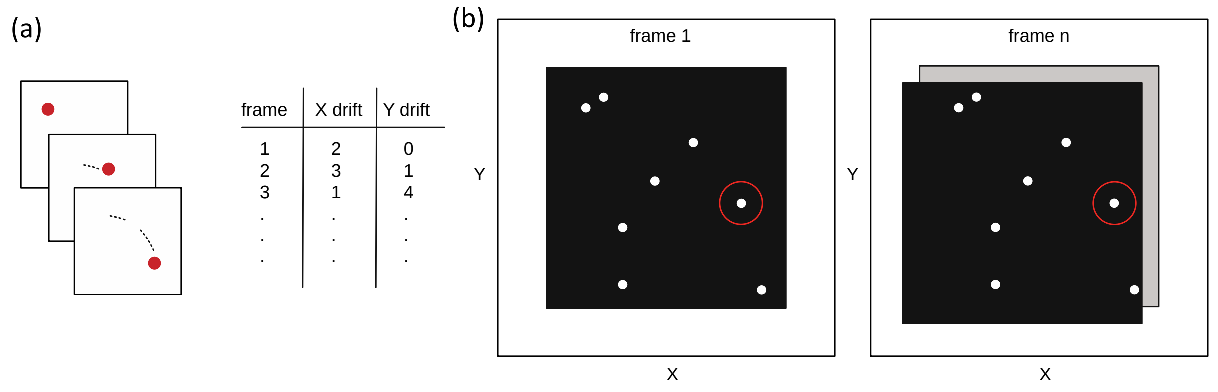

Supplementary Figure 19 – Single particle tracking and drift correction.

(a) Schematic representation (left) of the single particle tracking methodology used. By averaging the movement of all irreversibly bound liposomes simultaneously, a table of x and y displacement for each frame can be generated (right). (b) Using the generated table of drift, each frame in a given experiment can be replaced accordingly, thus removing any stage drift. To do so, the first frame (512 by 512 pixels) is placed in the middle of a significantly larger image (562 by 562 pixels) (left). Each consecutive frame from the experiment is then moved within the larger image (right), thus maintaining the absolute position of liposomes, here indicate by red circle. Iterating through each experiment, a new video is obtained where liposomes no longer move. A video to visualize this process can be found as Supplementary Video 1. By localizing each liposome in the first frame, the intensities can now reliably be extracted.

a)

b)

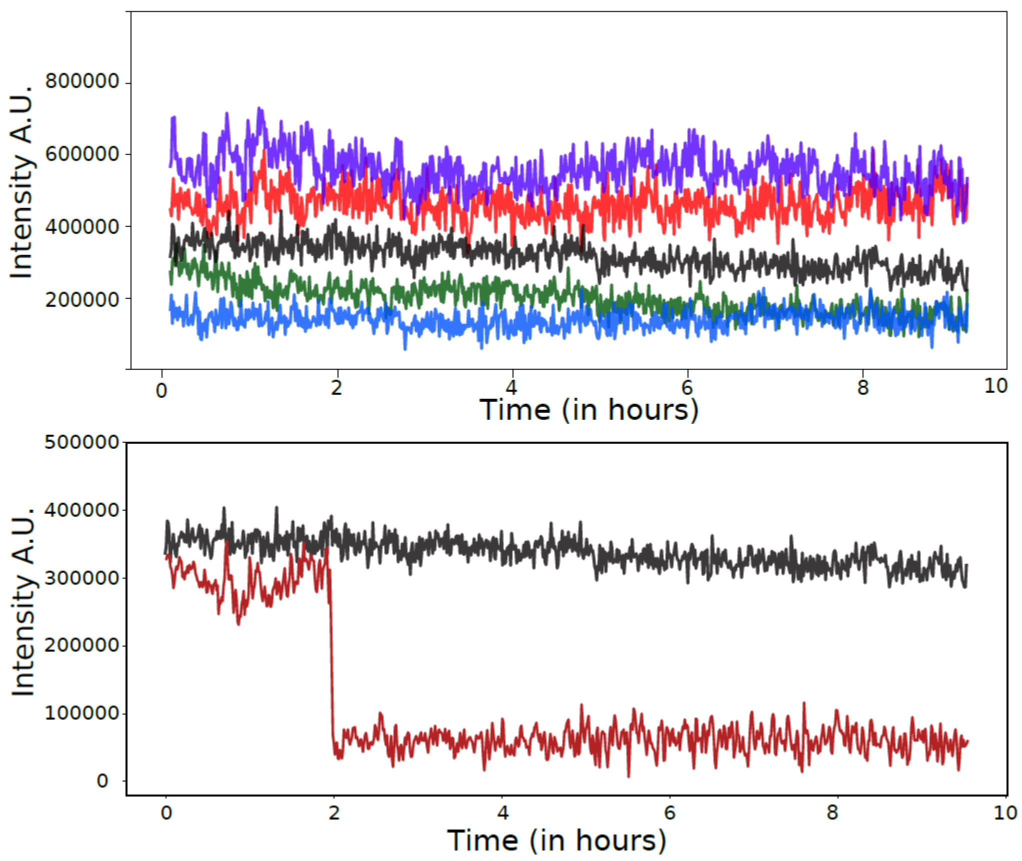

Supplementary Figure 20 – Control showing no significant photobleaching of ATTO 655 in the experimental time frame

The figure shows intensity traces for ATTO 655 loaded SUV’s recorded over a time period of 9.6h with a temporal resolution of 4.6s. (a) The photobleaching under these imaging conditions is negligible. (b) Comparing the stable non-leaking SUV’s (black) to the DNA nanopore induced leaking SUVs (red) confirms the negligible effect of photobleaching in our results.

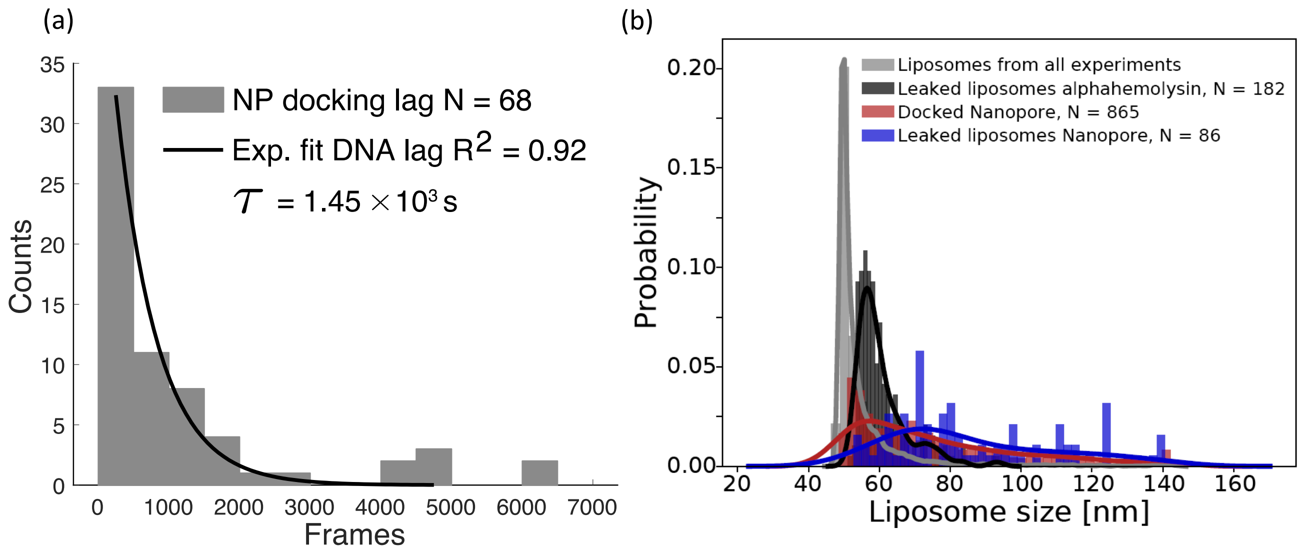

Supplementary Figure 21 – Study of nanopore docking, insertion and size dependence.

(a) Histogram of the docking lag, which shows a colocalization time of $\tau_1 = 1450$ seconds. (b) Liposome size distributions for i) all liposomes ii) liposomes with dye efflux s due to α-hemolysin iii) liposomes where a DNA nanopore is found to dock and iv) liposomes with dye efflux due to a DNA nanopore penetration. Liposome sizes based on calculations from encapsulation efficiency (Supplementary Fig. 30). The distributions for docked and leak liposomes due to DNA nanopores are shifted towards bigger liposome sizes, indicating a preference for smaller curvatures. The majority (80%) of the SUVs exhibited no detectable nanopore docking or ATTO transport, which combined with a single docking event on the blue channel strongly support the presence of a single nanopore docking on each individual SUV.

All liposomes tracked for DNA nanopore: 4412 liposomes

Total registered insertions: 86

All liposomes tracked for alpha hemolysin: 400 liposomes

Total registered insertions: 182

Supplementary Table 1 – Statistics on size distributions for both Alpha-Hemolysin and DNA Nanopore experiments.

Liposomes sizes where DNA nanopore successfully form pores

Liposome selection	Diameter size [nm]*	Membrane tension [$mN{\cdot}m^{-1}$]*
Median	78.37 $\pm$ 35.91	48.73 $\pm$ 22.60
Max	139.30 $\pm$ 0.78	27.40 $\pm$ 1.87
Min	52.18 $\pm$ 0.28	73.25 $\pm$ 4.99

Liposomes size information where alpha hemolysin successfully forms pores.

Liposome selection	Diameter size [nm]*	Membrane tension [$mN{\cdot}m^{-1}$]*
Median	57.67 $\pm$ 10.26	66.25 $\pm$ 12.64
Max	92.89 $\pm$ 0.52	41.10 $\pm$ 2.80
Min	50.71 $\pm$ 0.27	75.38 $\pm$ 5.14

* Provided errors is given as standard deviation

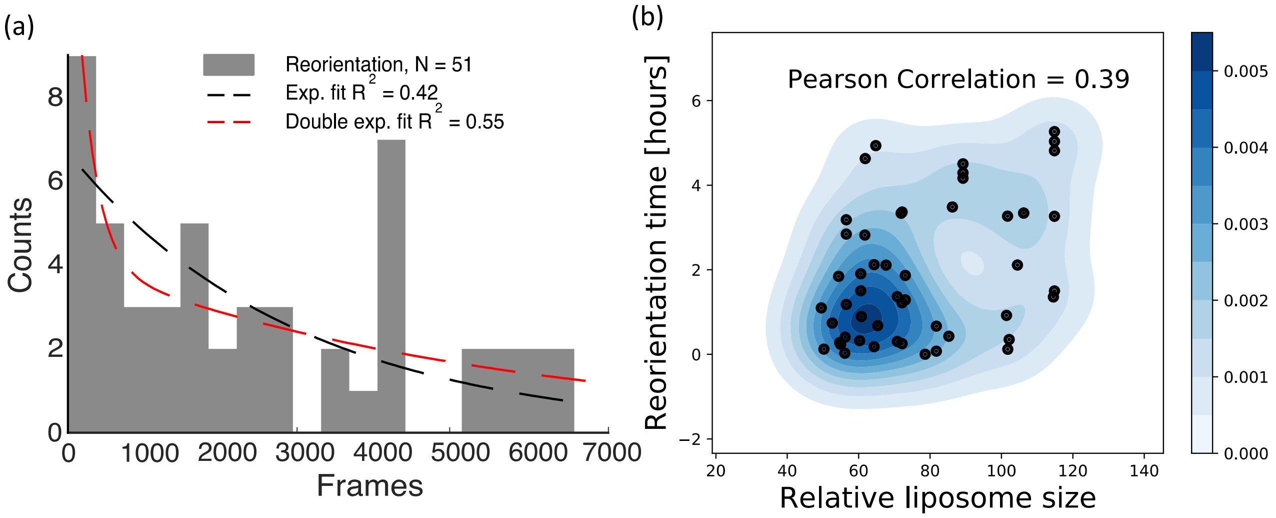

Supplementary Figure 22 – Reorientation and pore formation kinetics upon binding to SUVs.

(a) Histogram of the observed reorientation lags times for the DNA nanopores inserted, τ_2 showing insertion not to be a single step process (b) Distribution of reorientation and its dependence to liposome size. Reorientation time is positively correlated to the size of the liposome with a Pearson correlation coefficient at 0.39 and a p-value of 0.004. This supports that when the DNA nanopore is docked, it reorients faster on liposomes with smaller sizes and higher curvature.

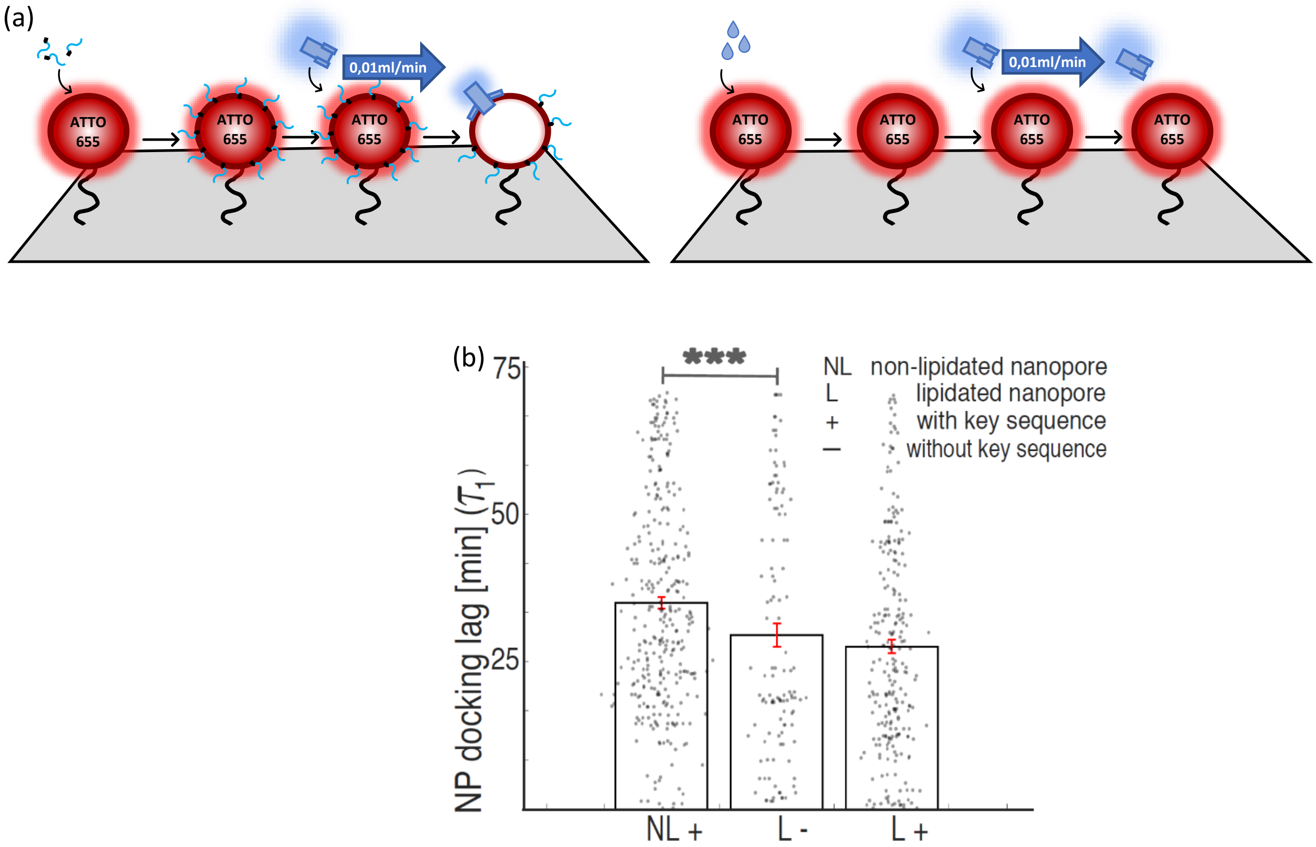

Supplementary Figure 23 – TIRF key experiments.

(a) Schematic setup of TIRF studies of SUV target insertion directed by lipidated DNA key oligos. Left, setup with keys flowed in (+) or right, control setup (-). Lipid anchored key strands was flowed into the immobilized SUVs and equilibrated for 30 min before 3x full chamber rinsing. Following, a continues flow of DNA nanopores with (L) or without (NL) lipidation was setup. Flow of nanopores in TIRF solution with a continuous rate of 0.01 ml/min for 50 min. (b) Nanopore colocalization lag time in setup. Asterisks (***) indicate a p-value of $<10^{-5}$ based on a two-sample t-test, showing prior lipidation of structures lower docking time significantly, while error bars show the standard error of the mean. No significant difference is found between key-decorated (+) and plain (-) SUVs with lipidated structures (p-value = 0.212).

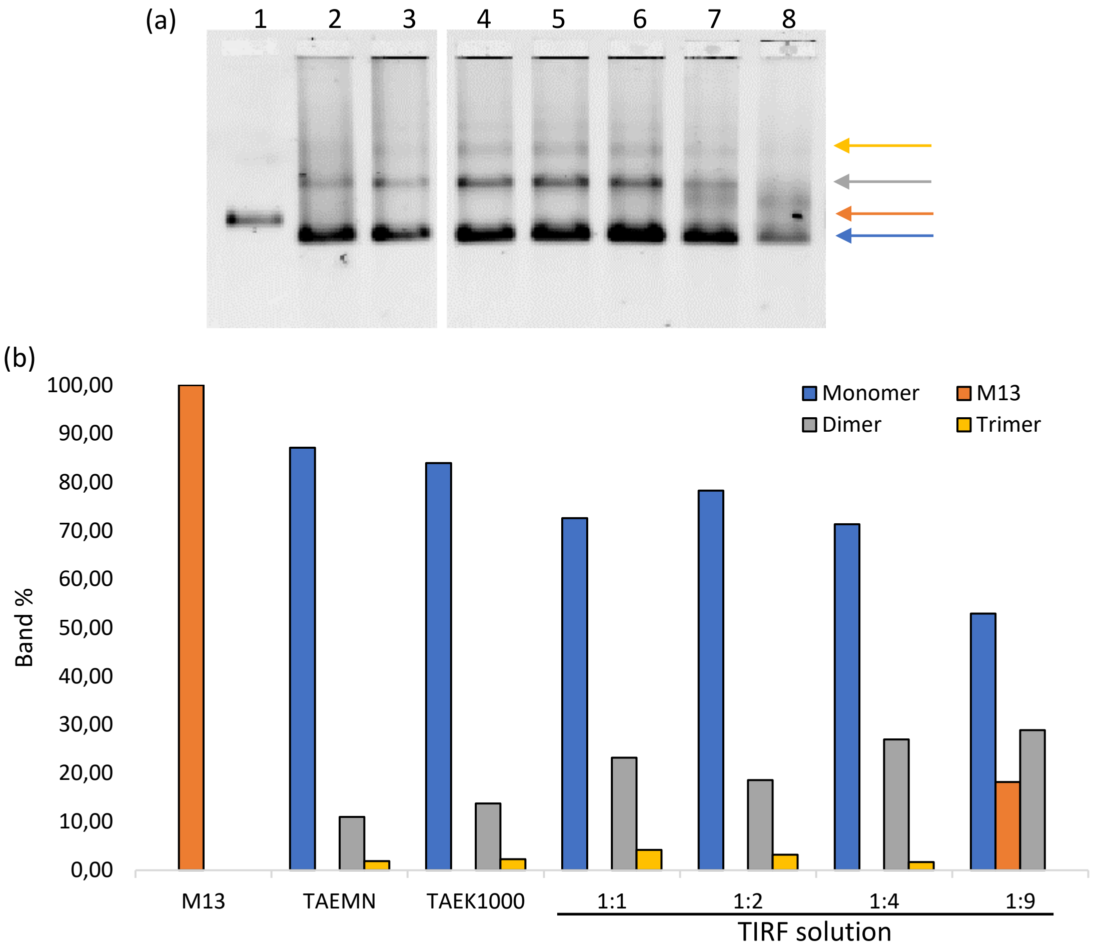

Supplementary Figure 24 – Stability of DNA nanopore in TIRF solution.

(a) 1 % AGE of DNA nanopores incubated in TIRF solution for 26 hours. In TIRF setup, DNA nanopores (in TAEMN) is incubated in TIRF buffer (400 mM Sucrose, 200 mM KCl) in a 1:4 mixture for up to 9 hours. Lane 1: M13. Lane 2: DNA nanopore (NP) in 1x TAEMN (1:1). Lane 3: NP in $TAEK_{1000}$ (1:1). Lane 4-8: NP in TIRF buffer of 1:1, 1:2, 1:4, and 1:9 dilution. (b) Gel quantification of the indicated bands. A good stability in TIRF setup conditions (1:4) during the 26 hours is observed. Even at 1:9 dilution for 26 hours DNA nanopores is still detectable.

a)

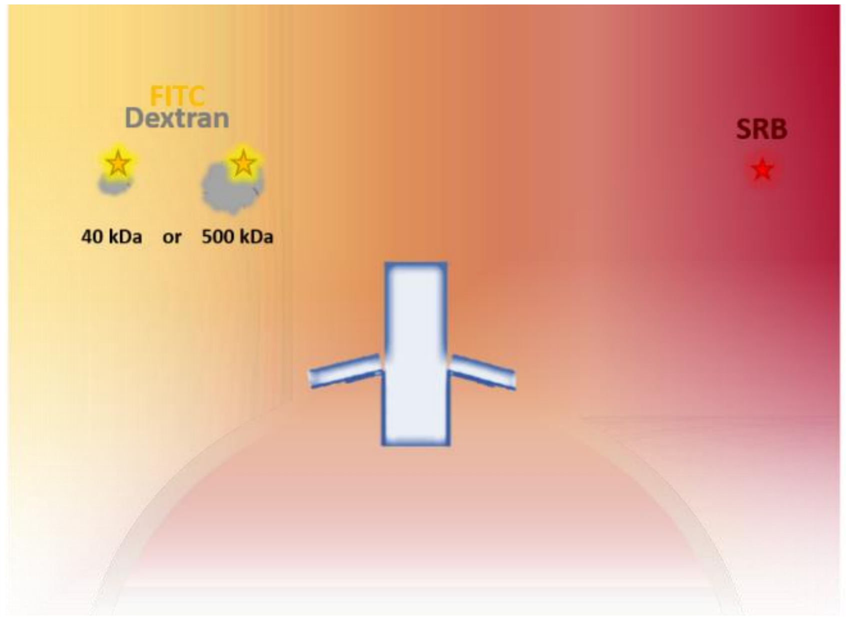

b)

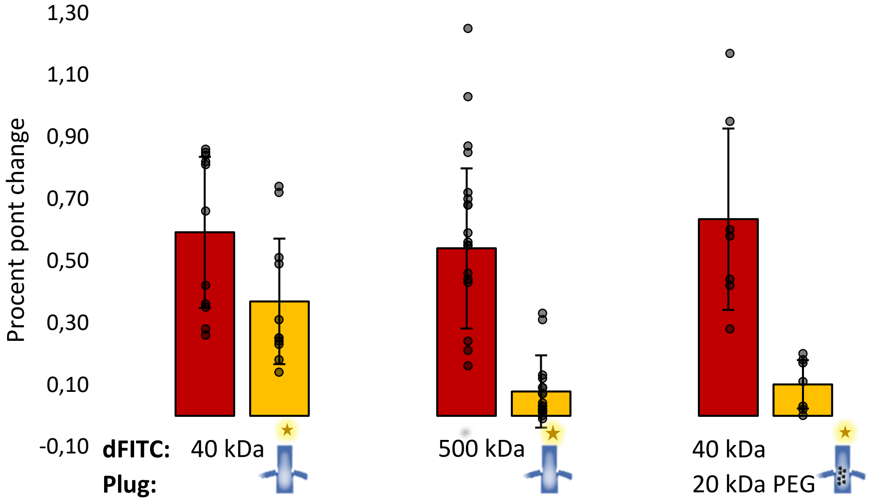

Supplementary Figure 25 – Confocal setup.

(a) Schematic of the CLSM setup of dyes included with wither small (left) or large (right) dFITC together with SRB dye. (b) Summary of insertions from frame -1 to 0 (filling) based on percent point change from the insertions depicted in Figure 4. Error bars show the standard deviation.

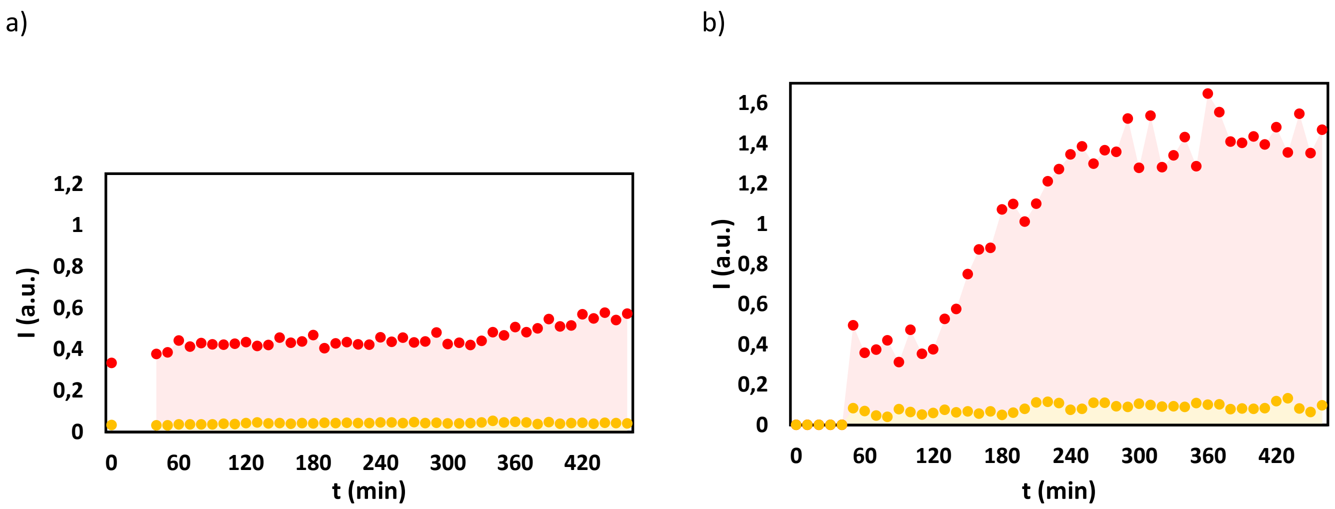

Supplementary Figure 26 – Non-filled and leaky CLSM traces of SRB and 40kDa dextran-FITC inside GUVs.

Representative traces of non-filled (a) and leaky (b) GUVs observed in CLSM influx assay with SRB and FITC-dextran(40kDa). (a) Non-filled traces show no internalization of SRB or FITC-dextran while the GUV bilayer remains intact. (b) Leaky GUV traces show a slow SRB dye increase behavior over extended periods of time, while FITC-dextran does not enter. Such traces were not included in the analysis as only single frame large SRB influx observations was used.

Supplementary Table 2 – CLSM normalized intensities of internal color (dFITC-40k)

SRB traces

Frame	T1	T2	T3	T4	T5	T6	T7	T8	T9	T10	T11	Avg	s.d.
-4	0,15	0,21	0,18	0,58	0,56	0,54	0,21	0,21	0,36	0,16	0,76	0,36	0,20
-3	0,18	0,36	0,18	0,55	0,53	0,54	0,19	0,22	0,40	0,18	0,73	0,37	0,19
-2	0,19	0,33	0,18	0,52	0,53	0,49	0,21	0,26	0,46	0,17	0,76	0,37	0,18
-1	0,33	0,29	0,18	0,58	0,53	0,53	0,21	0,20	0,52	0,17	0,85	0,40	0,21
0	1,14	1,14	1,02	0,84	0,95	0,89	0,87	1,06	0,87	0,99	1,13	0,99	0,11
1	0,97	1,04	1,05	0,81	0,98	0,99	0,84	1,11	0,92	1,08	1,15	0,99	0,10
2	1,03	1,14	1,03	0,83	0,98	0,91	0,85	1,07	0,87	1,22	1,24	1,02	0,14
3	0,98	1,04	1,01	0,80	1,03	0,92	0,86	1,02	0,84	0,95	1,23	0,97	0,11
4	1,05	0,98	1,02	0,84	1,02	0,92	0,85	1,03	0,89	0,98	1,16	0,98	0,09
5	1,02	0,99	1,03	0,83	1,07	0,98	0,88	1,00	0,90	1,01	1,22	0,99	0,10
6	1,00	1,05	0,99	0,83	1,02	0,96	0,89	0,99	0,92	1,03	1,19	0,99	0,09

FITC traces

Frame	T1	T2	T3	T4	T5	T6	T7	T8	T9	T10	T11	Avg	s.d.
-4	0,03	0,03	0,03	0,03	0,04	0,07	0,05	0,05	0,05	0,06	0,05	0,04	0,01
-3	0,04	0,04	0,03	0,03	0,04	0,06	0,05	0,04	0,04	0,07	0,05	0,05	0,01
-2	0,04	0,03	0,03	0,03	0,04	0,06	0,05	0,04	0,04	0,06	0,05	0,04	0,01
-1	0,04	0,03	0,03	0,03	0,04	0,06	0,05	0,04	0,04	0,06	0,06	0,04	0,01
0	0,22	0,26	0,52	0,17	0,55	0,31	0,30	0,78	0,28	0,37	0,78	0,41	0,21
1	0,30	0,26	0,64	0,19	0,68	0,29	0,41	0,92	0,33	0,49	0,92	0,49	0,25
2	0,33	0,27	0,70	0,23	0,76	0,33	0,44	0,92	0,35	0,57	0,96	0,53	0,25
3	0,36	0,27	0,74	0,26	0,79	0,33	0,49	0,92	0,38	0,50	0,98	0,55	0,25
4	0,39	0,28	0,77	0,29	0,82	0,31	0,52	0,91	0,40	0,52	0,99	0,56	0,25
5	0,40	0,28	0,79	0,32	0,84	0,32	0,55	0,91	0,41	0,53	1,01	0,58	0,25
6	0,43	0,27	0,79	0,32	0,86	0,33	0,56	0,91	0,43	0,55	0,98	0,58	0,24

The 11 traces observed of GUV dye 1-frame filling observed at normalized frame 0 used for Figure 4d. There is 10 minutes between each frame. The listed values are the internal intensity normalized to the outer intensity to observe the relative intensity increase.

Supplementary Table 3 – CLSM normalized intensities of internal color (dFITC-500k)

SRB traces

Frame	T1	T2	T3	T4	T5	T6	T7	T8	T9	T10	T11	T12	T13	T14	T15	T16	T17	T18	Avg	s.d.
-4	N/A	0,33	N/A	0,35	0,12	0,12	0,14	0,80	0,11	0,38	0,60	0,15	0,49	0,26	0,35	0,14	0,32	0,19	0,30	0,19
-3	N/A	0,38	N/A	0,32	0,12	0,17	0,19	0,88	0,12	0,38	0,63	0,16	0,53	0,27	0,36	0,14	0,29	0,20	0,32	0,20
-2	0,29	0,48	0,31	0,36	0,10	0,16	0,22	0,88	0,12	0,41	0,65	0,19	0,56	0,28	0,37	0,13	0,27	0,18	0,33	0,21
-1	0,50	0,53	0,50	0,38	0,11	0,17	0,28	0,79	0,12	0,37	0,67	0,17	0,58	0,27	0,34	0,15	0,31	0,20	0,36	0,20
0	0,71	1,09	0,74	1,10	0,66	0,72	1,15	0,95	1,15	0,96	1,13	1,42	1,01	0,95	1,02	1,00	1,01	0,64	0,97	0,20
1	0,80	0,95	0,78	1,15	0,73	0,67	1,21	0,90	1,16	0,99	1,09	1,32	0,96	1,03	1,12	1,04	0,99	0,59	0,97	0,19
2	0,81	0,97	0,90	1,14	0,77	0,68	1,15	0,84	1,26	0,97	1,14	1,11	0,99	1,05	1,08	1,03	0,99	0,58	0,97	0,17
3	0,81	1,00	0,90	1,12	0,70	0,66	1,13	0,86	1,20	1,02	1,12	1,12	0,94	1,04	0,97	1,09	1,03	0,58	0,96	0,17
4	0,86	0,98	0,92	1,05	0,69	0,66	1,15	0,91	1,13	0,98	N/A	1,12	0,91	0,99	1,04	1,03	1,04	0,57	0,94	0,16
5	0,86	1,03	0,87	0,99	0,63	0,70	1,14	0,85	1,18	1,00	N/A	1,11	0,92	1,08	1,05	1,02	1,01	0,59	0,94	0,17
6	0,85	0,99	0,94	1,03	0,64	0,65	1,12	0,84	1,17	1,06	N/A	1,19	0,91	1,01	1,07	1,06	1,04	0,60	0,95	0,18

FITC traces

Frame	T1	T2	T3	T4	T5	T6	T7	T8	T9	T10	T11	T12	T13	T14	T15	T16	T17	T18	Avg	s.d.
-4	N/A	0,04	N/A	0,09	0,03	0,03	0,07	0,04	0,04	0,03	0,04	0,06	0,03	0,04	0,07	0,03	0,06	0,03	0,04	0,02
-3	N/A	0,04	N/A	0,07	0,03	0,03	0,09	0,04	0,04	0,03	0,04	0,06	0,02	0,04	0,06	0,03	0,06	0,04	0,05	0,02
-2	0,04	0,05	0,04	0,09	0,03	0,03	0,09	0,03	0,04	0,04	0,04	0,07	0,03	0,04	0,07	0,03	0,08	0,04	0,05	0,02
-1	0,03	0,05	0,04	0,08	0,03	0,02	0,10	0,04	0,05	0,04	0,05	0,06	0,02	0,04	0,07	0,03	0,06	0,03	0,05	0,02
0	0,04	0,12	0,04	0,15	0,05	0,04	0,09	0,05	0,38	0,07	0,36	0,18	0,11	0,13	0,10	0,16	0,10	0,04	0,12	0,10
1	0,05	0,13	0,05	0,15	0,04	0,04	0,09	0,03	0,43	0,09	0,38	0,16	0,12	0,15	0,15	0,17	0,14	0,05	0,13	0,11
2	0,04	0,14	0,05	0,14	0,05	0,04	0,11	0,03	0,47	0,10	0,36	0,16	0,12	0,15	0,16	0,18	0,15	0,04	0,14	0,11
3	0,04	0,16	0,04	0,15	0,04	0,03	0,10	0,04	0,50	0,10	0,37	0,16	0,12	0,16	0,16	0,18	0,16	0,04	0,14	0,12
4	0,05	0,17	0,05	0,15	0,04	0,03	0,11	0,05	0,51	0,11	N/A	0,19	0,13	0,17	0,17	0,21	0,16	0,04	0,14	0,11
5	0,05	0,18	0,04	0,14	0,04	0,03	0,12	0,04	0,54	0,11	N/A	0,18	0,13	0,19	0,18	0,20	0,18	0,04	0,14	0,12
6	0,05	0,18	0,05	0,13	0,04	0,03	0,12	0,05	0,54	0,12	N/A	0,18	0,12	0,20	0,19	0,21	0,20	0,05	0,14	0,12

The 18 traces observed of GUV dye 1-frame filling observed at normalized frame 0 used for Figure 4h. There is 10 minutes between each frame. The listed values are the internal intensity normalized to the outer intensity to observe the relative intensity increase. N/A values indicate frames where the values were not possible to determine as these frames was before or after CLSM acquisition.

Supplementary Table 4 – CLSM normalized intensities of internal color (dFITC-40k + plug)

SRB traces

Frame	T1	T2	T3	T4	T5	T6	T7	Avg	s.d.
-4	0,35	0,17	N/A	N/A	0,82	0,44	0,34	0,42	0,22
-3	0,40	0,24	N/A	N/A	0,84	0,47	0,33	0,45	0,21
-2	0,39	0,18	N/A	N/A	0,80	0,46	0,46	0,46	0,20
-1	0,53	0,13	0,32	0,36	0,72	0,54	0,37	0,42	0,17
0	1,48	1,30	0,90	0,78	1,00	0,98	0,97	1,06	0,23
1	1,15	1,00	1,04	0,76	0,94	1,04	1,14	1,01	0,12
2	1,13	1,27	0,99	0,69	0,86	1,06	1,12	1,02	0,18
3	1,18	1,08	0,98	0,75	0,92	1,17	1,17	1,04	0,15
4	1,12	1,11	1,02	0,68	0,91	1,17	1,11	1,02	0,16
5	1,37	1,00	0,98	0,76	0,87	1,14	1,06	1,02	0,18
6	1,17	0,97	0,98	0,86	0,88	1,18	1,07	1,02	0,12

FITC traces

Frame	T1	T2	T3	T4	T5	T6	T7	Avg	s.d.
-4	0,07	0,06	N/A	N/A	0,04	0,06	0,16	0,08	0,04
-3	0,11	0,08	N/A	N/A	0,05	0,06	0,13	0,09	0,03
-2	0,09	0,07	N/A	N/A	0,06	0,04	0,11	0,07	0,02
-1	0,07	0,06	0,09	0,09	0,04	0,05	0,16	0,08	0,04
0	0,25	0,26	0,20	0,09	0,06	0,08	0,33	0,18	0,10
1	0,33	0,22	0,21	0,12	0,04	0,07	0,33	0,19	0,11
2	0,47	0,27	0,32	0,11	0,04	0,10	0,28	0,23	0,14
3	0,39	0,38	0,18	0,12	0,06	0,12	0,31	0,22	0,13
4	0,36	0,44	0,21	0,19	0,05	0,14	0,31	0,24	0,12
5	0,35	0,39	0,22	0,17	0,05	0,16	0,31	0,24	0,11
6	0,36	0,43	0,19	0,14	0,06	0,17	0,31	0,24	0,12

The 7 traces observed of GUV dye 1-frame filling observed at normalized frame 0 used for Figure 4l. There is 10 minutes between each frame. The listed values are the internal intensity normalized to the outer intensity to observe the relative intensity increase. N/A values indicate frames where the values were not possible to determine as these frames was before or after CLSM acquisition.

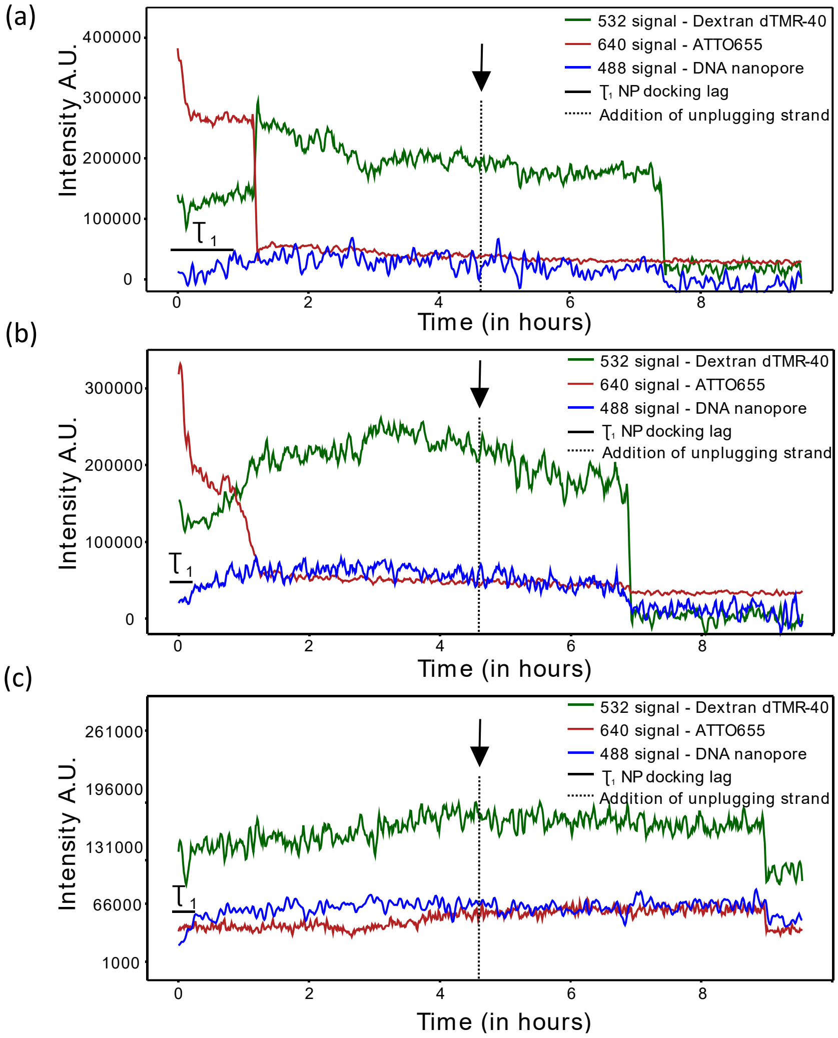

Supplementary Figure 27 – Real-time sensing of "unplugging" oligonucleotide.

Real-time sensing of unplugging of DNA nanopore insertion into ATTO 655 and 40 kDa dextran-tetramethyl rhodamine (dTMR-40k) encapsulated SUVs. (a) and (b) shows 2 representative sequentially leaking liposomes, where the small ATTO 655 dyes translocate through the inserted and plugged DNA nanopore. Note that due to spectral overlap of ATTO 488 with TMR, the signal of individual nanopore docking on TMR loaded SUVs resulted in small signal in the blue channel. 4.4 hours into the experiment the "unplugging" strand is added, opening the nanopore and allowing translocation of the larger dTMR-40k. (c) If docking/penetration of the DNA nanopore occurs after addition of the "unplugging" strand the two dye molecules are translocating simultaneously. This behavior is observed two times.

Supplementary Table 5 – Registered dye flow events in real-time sensing assay sorted by event type.

Event (pre/post addition of "unplugging strand)	# Recorded events
ATTO 655 flow only (pre)	125
ATTO 655 flow only (post)	3
ATTO 655 flow (pre) and dTMR-40k flow (pre)	1
ATTO 655 flow (pre) and dTMR-40k (post)	5
ATTO 655 and dTMR-40 simultaneous flow (pre)	0
ATTO 655 and dTMR-40 simultaneous flow (post)	2

A total of 3174 SUV's have been investigated for the real-time sensing experiment. It is found that 128 SUVs shows translocation of ATTO655 which corresponds to an efficiency of 4%. 5 cases demonstrate a successful sensing by unzipping the plugs. In 2 cases the two dye molecules are found to translocate simultaneously post addition of "unplugging" strand. Presumably, the single outlier for which dTMR-40k release is observed already before the pore is unplugged corresponds to a pore, which has only partially been plugged in the first place.

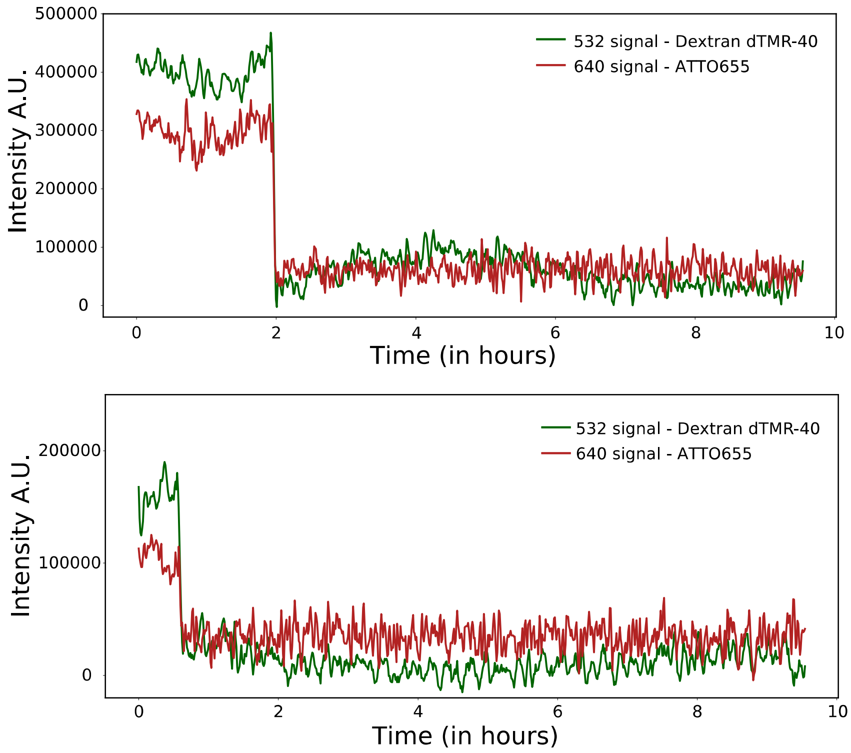

Supplementary Figure 28 – Control for real-time sensing with non-plugged DNA nanopores.

Control sensing experiment with non-plugged DNA nanopores and ATTO 655 and 40 kDa dextran-tetramethyl rhodamine (dTMR-40k) encapsulated SUVs. The inner pore of the DNA nanopore without plug is as reported 9nm which allows both ATTO 655 and dTMR-40k to translocate.

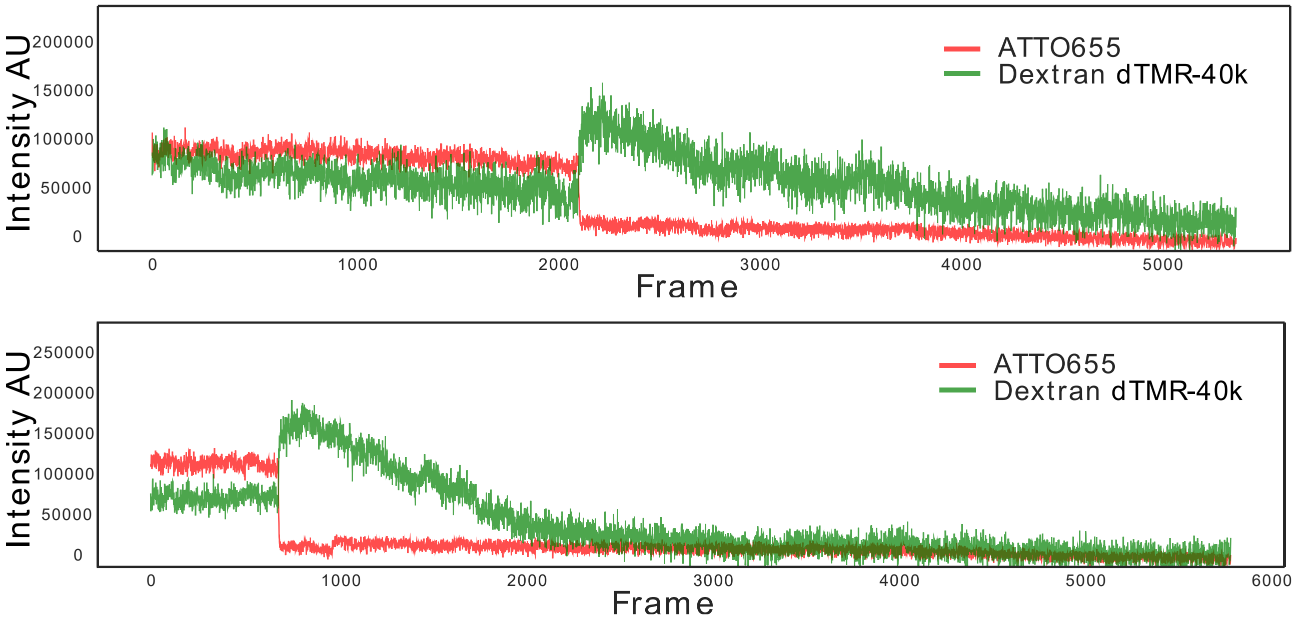

Supplementary Figure 29 – Control for real-time efflux of ATTO 655 via the α-hemolysin pore.

Representative traces from control experiment for real-time specific efflux of the small ATTO 655 through the narrow α-hemolysin pore. 100 µmol α-hemolysin is flowed into the surface with immobilized ATTO 655 and 40 kDa dextran-tetramethyl rhodamine (dTMR-40k) encapsulated SUVs. A total of 244 SUV’s are tracked and investigated and 97 (40%) of these show translocations of ATTO 655 as shown in the figure. ATTO 655 translocation results in dTMR-40k signal increase due to FRET between the two chromophores when they are entrapped together in the SUVs (The TMR signal increase can also be seen after ATTO 655 efflux with the plugged DNA nanopore in Supplementary Fig. 27). The TMR bleaching seems faster after ATTO 655 translocation, probably because the absorbed energy in dTMR-40k can no longer be transferred which results in a higher intensity signal and more photobleaching.

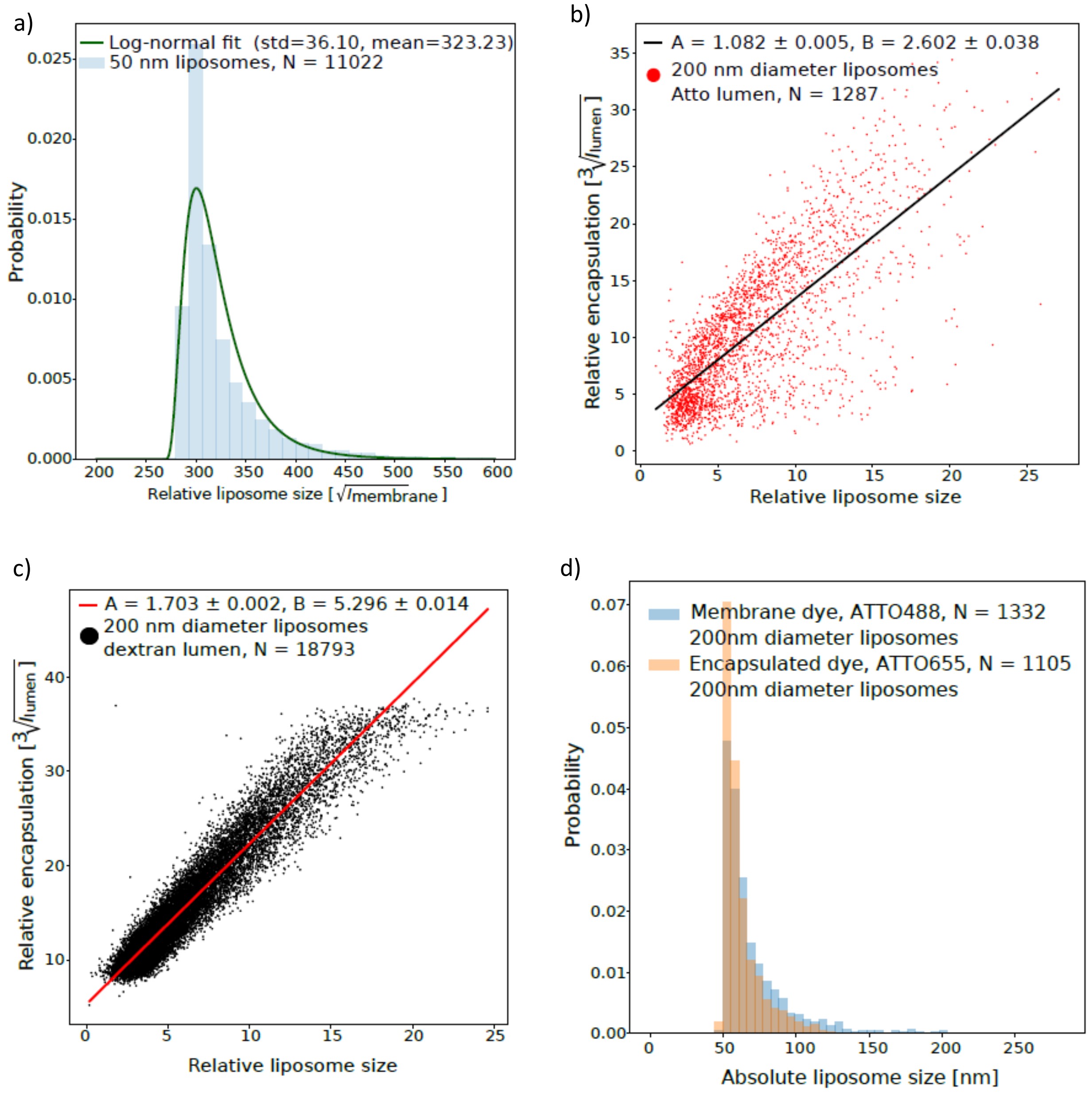

Supplementary Figure 30 – Loading efficiency of various dye encapsulated SUVs.

Histograms of size distributions of liposomes labeled on membrane with lipidated ATTO 488 and data recorded with identical settings as throughout the paper as to correlate membrane intensity to size calibration was done following our well-established methodology[7].

(a) Liposome extruded around 50 nm. The distribution follows a clear log-normal distribution. From here the mean integrated square root of the intensity from the membrane stained dye is found which is used to convert all membrane intensities to absolute sizes[7]. (b-c) Quantification of the amount of (b) ATTO 655 and (c) 40 kDa dextran-tetramethyl rhodamine (dTMR-40k) encapsulated for all liposome sizes. A linear dependence of the third root of the integrated lumen intensity with liposome sizes as shown earlier[8]. This conversion is found to be $S = A_{corr} \cdot \sqrt[3]{I_{lumen}} + B_{corr},\ \ A_{corr} = 1.049 \pm 0.006,\ B_{corr} = 2.671 \pm 0.055$ for ATTO655 encapsulated (b) and $A_{corr} = 1.703 \pm 0.002,\ B_{corr} = 5.296 \pm 0.014$ for dTMR-40k encapsulated (c). The linear fit allows to extract liposome sizes based on the encapsulated dye. (d) Similar Size distribution of liposomes is obtained when calculated by their membrane dye[7] or their encapsulated dye[8].

Supplementary Note 3 – Numerical relation between observed flow rate and the pore size described by the Hagen-Poiseuille relation.

In order to gain further insights in translocation of molecules through a transmembrane pore, we have investigated the numerical relation between the pore diameter and the observed flow rate. As a simplification the diffusion can be approximated with a flow that follows the Hagen-Poiseuille relation.
For the 1.4nm wide alpha hemolysin pore we measured a flow rate of 0.014 ± 0.010 l/s. For the 9.6nm wide DNA nanopore we measured a flow rate of 0.098 ± 0.087 l/s. We report a 7.0 ± 8.1-fold increase in flow rate (error bars correspond to one standard deviation). The pore diameter of DNA nanopore on the other hand is 6.8-fold larger than the Hemolysin one (9).
The relation between pore size and flow can be described by the pressure difference in a Newtonian and incompressible fluid under laminar flow through a pore with constant cross section. The flux is given as

$$J = \frac{\Delta p}{32} \frac{{d_p}^2 \varepsilon}{x_p \mu_p} \tag{2}$$

Where Δp is the transmembrane pressure, d_p is the diameter of the pores, ε is the surface porosity of the liposome membrane, x_p is the length of the pore, and μ_p is the viscosity of the permeating fluid.
Assuming Δp, ε and μ_p are unchanged from the two experiments we get the relation:

$$\frac{{d_{NP}}^2}{x_{NP}} = \text{const.} \cdot \frac{{d_\alpha}^2}{x_\alpha} \tag{3}$$

$$\frac{(9.6 \cdot 10^{-9})^2}{35 \cdot 10^{-9}} = \text{const.} \cdot \frac{(14 \cdot 10^{-10})^2}{100 \cdot 10^{-10}}$$

$$\rightarrow \text{const} = \mathbf{13.43}$$

The theoretical 13.4-fold increase for the ATTO 655 flow though the DNA nanopore are thereby in relatively good agreement within the observed experimentally flow increase. This is under the approximation of the diffusion with a flow and despite a) the different pore charges which is not taken into consideration in the theoretical calculation, but might affect the efflux rates b) the pore diameter in Hemolysin is not constant along the channel tunnel (9) c) the fact the membrane curvature and appended tension varies the narrow point for SUVS of different membrane curvature (10)

Supplementary Note 4 – Freely diffusion model of the translocated dye molecules.

The diffusion of a dye molecule outside of the vesicle entails a) finding the pore following free diffusion and b) translocating across the pore following free diffusion and any potential mechanical hindrance.

The Stokes-Einstein equation describes diffusive processes of spherical particles in liquid that undergo Brownian motion, but it does not adequately describe the highly restricted motion within the pore nor the repulsive interactions of similarly charged species. So, we note it can only be used as an approximation.

The unrestricted diffusion of a particle is given by

$$D = \frac{k \cdot T}{6 \cdot \pi \cdot R_H \cdot \eta} \quad (4)$$

Where R_H is the hydrodynamic radius of the translocated molecule (here an ATTO 655 carboxy and a 40 kDa Dextran-tetramethyl rhodamine).

The root mean square displacement of the diffusive dye molecules within a time t in the 3 dimensions of the vesicle is given as:

$$\langle r^2 \rangle = 6Dt \quad (5)$$

The flow rate constant k and the half-life time τ are inverse correlated. This gives that the diffusion coefficient and the rate constant are proportional:

$$k \propto \frac{1}{\tau} \propto \frac{D}{r^2} \quad (6)$$

where r is the radius of the vesicle.
The half-life time for an ATTO 655 dye encapsulated within a vesicle with a radius of 35nm would thereby theoretically be expected to be $\tau \cong 3.3 \cdot 10^{-6}$sec. We would therefore expect that within microseconds, half of the dye molecules have searched the vesicle found the pore. The unhindered diffusion out of the pore is expected to occur in the same time scale. Our direct experimental observation of rates however shows efflux rates in the order of milliseconds to seconds (dTMR-40k translocating through the DNA nanopore of: 0.0057 $\pm$ 0.0036 I/s, while ATTO 655 flow rate was found to be 0.098 $\pm$ 0.087 I/s).
An explanation for this observed reduced translocation could be that the translocated molecules interact electrostatically with the pore or interact with the inner membrane of the vesicle, both of which could result in hindrance and therefore a delayed translocation.

Because we have used the same pore and membrane composition, we can assume that the effect of Pore and membrane characteristic are similar for the Atto-655 and dTMR-40k the and the relation between the observed and theoretical flow rates and pore size can be analyzed.
Comparison of flow rates yield a 17.09 $\pm$ 18.50-fold faster flow rate for translocation of the small ATTO 655 molecule (error bars correspond to one standard deviation). We note that the temporal resolution of the confocal experiments limits the accurate extraction of flow rates.

Using the Stokes-Einstein Kinetics we would expect:

$$D_{ATTO} = const \cdot D_{dextran} \quad (7)$$

$$\frac{k \cdot T}{6 \cdot \pi \cdot R_{H\,ATTO} \cdot \eta} = const \cdot \frac{k \cdot T}{6 \cdot \pi \cdot R_{H\,Dextran} \cdot \eta}$$

$$\frac{1}{R_{H\,ATTO}} = const \cdot \frac{1}{R_{H\,Dextran}}$$

$const = \mathbf{7.59}$ times higher flow rate for the ATTO 655, using a hydrodynamic radius of 5.86 Å for ATTO 655 and 44.5 Å for the 40 kDa Dextran-tetramethyl rhodamine. While Stoke-Einstein does not include the highly restricted motion within the pore nor the repulsive interactions of similarly charged species. The calculated rates relation is in relatively good agreement with the recorded values.

Supplementary Note 5 – Synthesis of Azido-palmitoyl building block ((S)-1-azido-3-(palmitoyloxy)propan-2-yl sulfate).

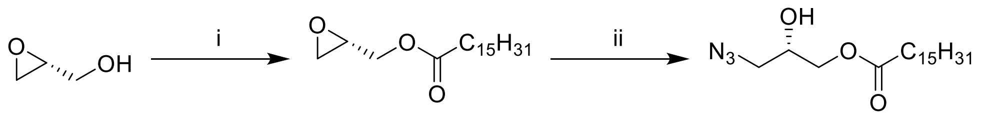

1 **2** **3**

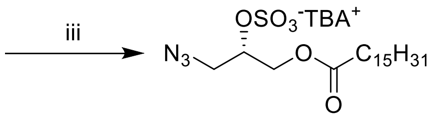

4

i) palmitoyl chloride, DMAP, DCM, r.t., 19h, 95%, ii) NaN_3, NH_4Cl, DMF, mw, 100°C, 15min, 88%, iii) a) pyridine-SO_3 complex, toluene, mw, 100°C, 20min, b) $Bu_4N^+OH^-$x30H_2O, H_2O, r.t., 1h, 87%.

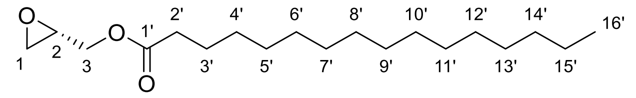

To a stirred solution of **1** (R)-(+)-glycidol (0.40 mL, 6.02 mmol, 1.0 eq) in dry dichloromethane (5 mL) under nitrogen atmosphere at 0 °C 4-dimethylaminopyridine (882 mg, 7.22 mmol, 1.2 eq) was added and stirred for 30 min. Palmitoyl chloride (2.2 mL, 7.25 mmol, 1.2 eq) was added dropwise and the cooling bath was removed. Stirring was continued for 19h at room temperature. The reaction mixture was filtered through a short pad of silica (dichloromethane) and the solvent was removed under reduced pressure. The crude product was purified by column chromatography (SiO_2, petroleum ether/ethyl acetate 6:1) yielding 1.79 g (5.73 mmol, 95 %) of **2** as a white solid.

R_f: 0.55 (petroleum ether/ethyl acetate 3:1); **^{1}H NMR** (400 MHz, $CDCl_3$): δ [ppm] = 0.86 (t, 3H, *J* = 6.8 Hz, 16'-H_3), 1.16-1.36 (m, 24H, 4'-H_2-15'-H_2), 1.62 (tt, 2H, *J* = 7.3, 7.6 Hz, 3'-H_2), 2.33 (t, 2H, *J* = 7.6 Hz, 2'-H_2), 2.63 (dd, 1H; *J* = 4.7, 2.6 Hz, 1-H_a), 2.82 (dd, 1H, *J* = 4.7, 4.7 Hz, 1-H_b), 3.15-3.21 (m, 1H, 2-H), 3.90 (dd, 1H, *J* = 12.3, 6.3 Hz, 3-H_a), 4.39 (dd, 1H, *J* = 12.3, 3.1 Hz, 3-H_b); **^{13}C NMR** (101 MHz, $CDCl_3$): δ [ppm] = 14.21 (C-16'), 22.80, 29.23, 29.36, 29.47, 29.56, 29.70, 29.76, 29.77, 29.78 (C-4'-C15'), 24.99 (C-3'), 34.18 (C-2'), 44.74 (C-1), 49.48 (C-2), 64.84 (C-3), 173.62 (C-1'); **MS** (ESI): 335.26 $[M+Na]^+$, 647.52 $[2M+Na]^+$; **HRMS** (ESI): calcd. for $C_{19}H_{36}NaO_3$ 335.2557 $[M+Na]^+$, found 335.2566.

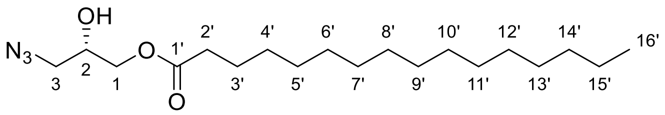

A suspension of **2** (200 mg, 0.64 mmol, 1.0 eq), sodium azide (125 mg, 1.92 mmol, 3.0 eq) and ammonium chloride (103 mg, 1.93 mmol, 3.0 eq) in dry DMF (3 mL) was stirred under microwave irradiation at 100 °C for 15 min. Water (10 mL) was added and the aqueous layer was extracted with ethyl acetate (3x 10 mL). The combined organic layers were washed with water (1x 10 mL) and dried over magnesium sulphate. The solvent was removed under reduced pressure and the residue was purified by column chromatography (SiO_2, petroleum ether/ethyl acetate 4:1) yielding 200 mg (0.56 mmol, 88 %) of **3** as white solid.

R_f: 0.36 (petroleum ether/ethyl acetate 3:1); **^{1}H NMR** (400 MHz, $CDCl_3$): δ [ppm] = 0.87 (t, 3H, *J* = 6.8 Hz, 16'-H_3), 1.18-1.36 (m, 24H, 4'-H_2-15'-H_2), 1.56-1.67 (m, 2H, 3'-H_2), 2.34 (t, 2H, *J* = 7.6 Hz, 2'-H_2), 2.61 (bs, 1H, OH), 3.37 (dd, 1H, *J* = 12.7, 6.4 Hz, 3-H_a), 3.41 (dd, 1H, *J* = 12.7, 4.7 Hz, 3-H_b), 3.96-4.03 (m, 1H, 2-H), 4.12 (dd, 1H, *J* = 11.9, 6.1 Hz, 1-H_a), 4.16 (dd, 1H, *J* = 11.9, 4.7 Hz, 1-H_b); **^{13}C NMR** (101 MHz, $CDCl_3$): δ [ppm] = 14.23 (C-16'), 22.81, 29.24, 29.36, 29.48, 29.57, 29.71, 29.76, 29.77, 29.79 (C-4'-C-15'), 25.01 (C-3'), 34.22 (C-2'), 53.62 (C-3), 65.62 (C-1), 69.26 (C-2), 174.13 (C-1'); **MS** (ESI): 378.27 $[M+Na]^+$, 733.55 $[2M+Na]^+$; **HRMS** (ESI): calcd. for $C_{19}H_{37}N_3NaO_3$ 378.2727 $[M+Na]^+$, found 378.2715.

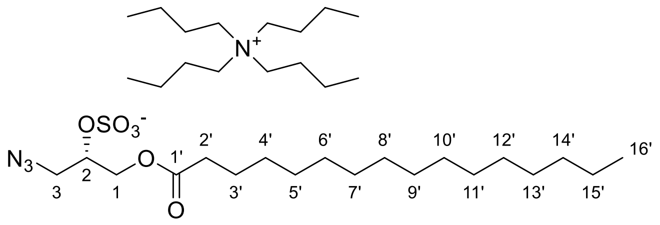

A mixture of **3** (200 mg, 0.56 mmol, 1.0 eq) and pyridine-sulfur trioxide complex (87 mg, 0.55 mmol, 1.0 eq) in dry toluene (5 mL) was stirred at 100 °C for 20 min under microwave irradiation. The mixture was treated with water (1 mL), stirred for 5 min at room temperature and subsequently tetrabutylammonium hydroxide 30-hydrate (448 mg, 0.56 mmol, 1.0 eq) was added. Stirring was continued for 1 h at room temperature, water (2 mL) was added and the aqueous layer was extracted with ethyl acetate (3x 5 mL). The combined organic layers were dried over sodium sulfate and the solvent was removed under reduced pressure yielding 332 mg (0.49 mmol, 87 %) of **4** as clear oil.

^{1}H NMR (400 MHz, $CDCl_3$): δ [ppm] = 0.84 (t, 3H, *J* = 6.8 Hz, 16'-H_3), 0.96 (t, 3H, *J* = 7.3 Hz, TBA-CH_3), 1.16-1.31 (m, 24H, 4'-H_2-15'-H_2), 1.40 (qt, 8H, *J* = 7.3, 7.3 Hz, TBA-$\mathit{CH_2}CH_3$), 1.50-1.66 (m, 10H, 3'-H_2, TBA-$NCH_2\mathit{CH_2}$), 2.22-2.33 (m, 2H, 2'-H_2), 3.16-3.27 (m, 8H, TBA-NCH_2), 3.46 (dd, 1H, *J* = 12.8, 4.6 Hz, 3-H_a), 3.66 (dd, 1H, *J* = 12.8, 5.3 Hz, 3-H_b), 4.23 (dd, 1H, *J* = 11.3, 6.3 Hz, 1-H_a), 4.28 (dd, 1H, *J* = 11.3, 4.6 Hz, 1-H_b), 4.62 (m, 1H, 2-H); **^{13}C NMR** (101 MHz, $CDCl_3$): δ [ppm] = 13.62 (TBA-CH_3), 14.09 (C-16'), 19.66 (TBA-$\mathit{CH_2}CH_3$), 22.66 (C-3'), 23.89 (TBA-$NCH_2\mathit{CH_2}$), 29.14, 29.27, 29.33, 29.45, 29.59, 29.62, 29.66, 31.89 (C-4'-C-15'), 34.12 (C-2'), 51.19 (C-3), 58.61 (TBA-NCH_2), 62.91 (C-1), 72.78 (C-2), 173.35 (C-1'); **MS** (ESI): 242.28 $[TBA]^+$, 434.22 $[M-TBA]^-$; **HRMS** (ESI): calcd. for $C_{19}H_{36}N_3O_6S$ 434.2330 $[M-TBA]^-$, found 434.2222.

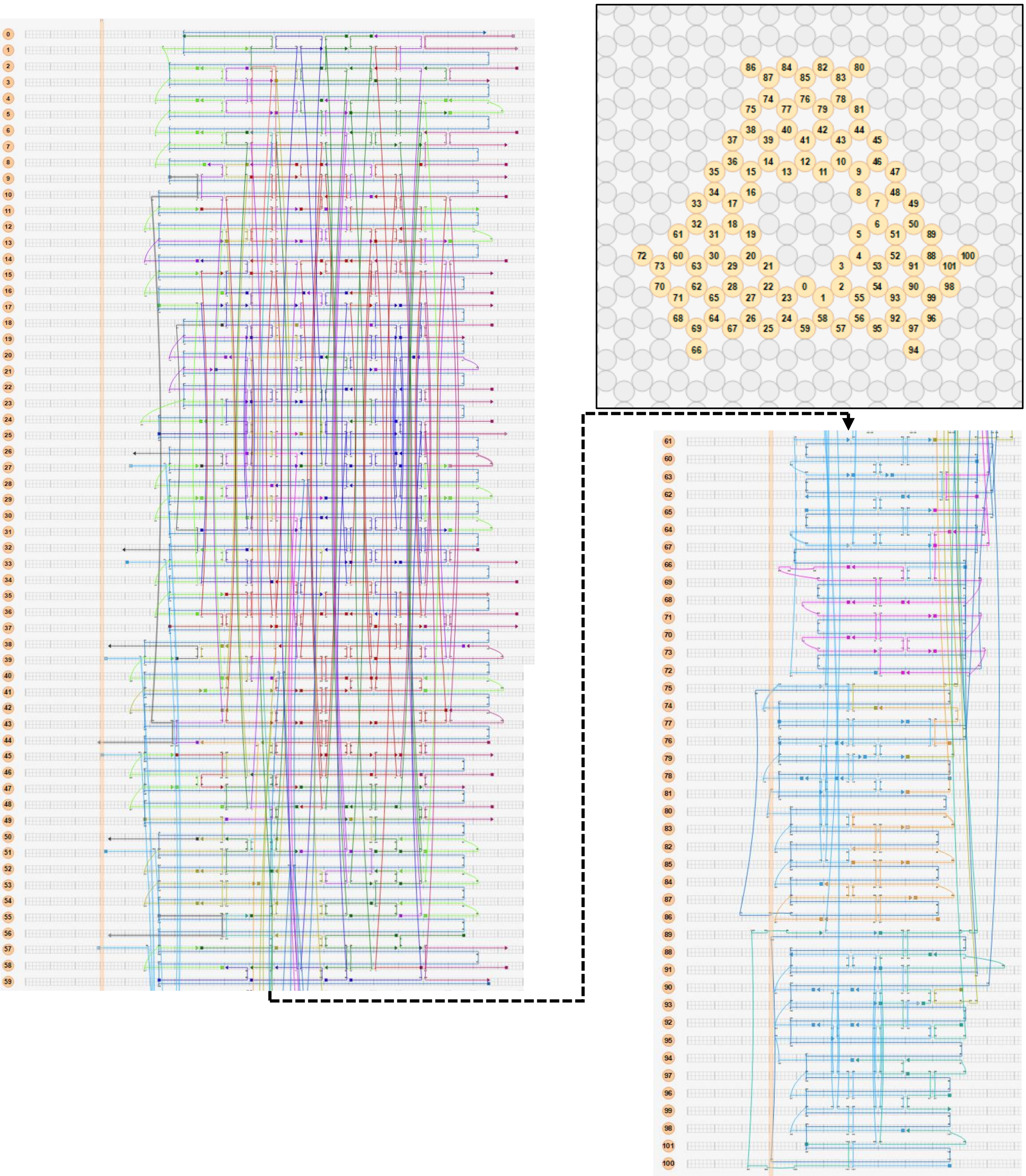

Supplementary Figure 31 – CaDNAno design of DNA nanopore.

CaDNAno blueprint of designed structure and with included top-view. The image is separated at the channel to flap transition.

Supplementary Note 6 – DNA origami staples.

A total of 219 staples are used for annealing the DNA nanopore (one of the “Top” sets, all “cyl”, “inner” and “flap” strands).

To create the lipidated structure, strands in the green module are used with a 3’ cholesterol or lipid functionalized anchor as described elsewhere.

For internal immobilization inner strands are elongated with handle strands inner -> inner (2) (blue module)

For TIRF of CLSM the 10 dye handle strands are added in excess to substitute top strands, purified and before addition of the fluorescent strand and a final purification.

Strands used for FRET are indicated below their original sequence with inserted IDT sequence.

Sequence-stretches of staples used for specific purposes other than structural use is highlighted in bold.

Supplementary Table 6 – DNA sequences.

Module	Notes	DNA Sequence
Top_A	a	TATGCAAAATTCGACAACTCGTATTAAATC**TACACTCA**
Top_A	a	**GCACATAC**ATTTTAGTTAATCAGTATAAAGCC
Top_A	a	CTAAAGGACGCCTGTCCTTGAAAACA**GGATCATA**
Top_A	a	**CTTGTCAT**TAGCGATAGCTTAGATTAAGACGCTGATTTTCCAAACATC
Top_A	a	CTGGTAATAGCGGATAAGTGC**ACTGCATA**
Top_A	a	**GATCAAAC**ACTAATAGTAGTTTTTAGAACCCT**TAGTAGAC**
Top_A	a	ACAGGGAAGTTTTCAATTTTATCCTGAATATTTTGCACCCAGCTA**GAACATTT**
Top_A	a	**ACCTTGTC**GTACAAACTACAAATTGCGAATAA**GAGGAACA**
Top_A	a	**GCTAGTAC**ACATGTTTTAAAGGCATCAATTCT
Top_A	a	**GAGGCTAA**CCCTCAATCAATATCTAACCTCACTGATAG
Top_A	a	**CAGTAAGC**ACAAGCAAGCCGTGGCGAGAAAGGAAGGGAA
Top_A	a	**CTCAGGTA**TAATGCAGAACGCACTCATCGAGA**GCTAGTAT**
Top_A	a	TCAAAAAGATTAAGAGGAATGCTGTAGCTCA**GATTAACG**
Top_A	a	**TCTCCATC**TGAGCAAAAGTTAAAGGCCGCTTTTTAATTTT
Top_A	a	**TCAGAATG**CGTCGAGAGGGTTGATATATTTTTCAAATAT**AAGTCATA**
Top_A	a	**CGGAATAC**CTTTGCCCGAAGCGGATTGCATTTTTTTTGCAAAAGA
Top_A	a	**TTGATACC**GTGGACTCCAACGTCATCCACTATTAAAGAAC**GATAACGA**
Top_A	a	**GATCTTGA**GTGTTTTTATAAAACAGGAAAAAC
Top_A	a	**AGCAACCG**GCTGCGCGTAACCACCACTACGCCAGAATCCTGAGAA**AGGATACC**
Top_A	a	**CACTTAGA**TTAATGCGCGAAAATATCAAA**TTACGATA**
Top_B	b	TATGCAAAATTCGACAACTCGTATTAAATC**TATGATCC**
Top_B	b	**TCTAAGTG**ATTTTAGTTAATCAGTATAAAGCC
Top_B	b	CTAAAGGACGCCTGTCCTTGAAAACA**TGAGTGTA**
Top_B	b	**TTAGCCTC**TAGCGATAGCTTAGATTAAGACGCTGATTTTCCAAACATC
Top_B	b	CTGGTAATAGCGGATAAGTGC**GTCTACTA**
Top_B	b	**CATTCTGA**ACTAATAGTAGTTTTTAGAACCCT**TATGCAGT**
Top_B	b	ACAGGGAAGTTTTCAATTTTATCCTGAATATTTTGCACCCAGCTA**TCGTTATC**
Top_B	b	**GTACTAGC**GTACAAACTACAAATTGCGAATAA**CGTTAATC**
Top_B	b	**GACAAGGT**ACATGTTTTAAAGGCATCAATTCT
Top_B	b	**ATGACAAG**CCCTCAATCAATATCTAACCTCACTGATAG
Top_B	b	**GGTATCAA**ACAAGCAAGCCGTGGCGAGAAAGGAAGGGAA
Top_B	b	**TCAAGATC**TAATGCAGAACGCACTCATCGAG**AGGTATCCT**
Top_B	b	TCAAAAAGATTAAGAGGAATGCTGTAGCTCA**TGTTCCTC**
Top_B	b	**GTATTCCG**TGAGCAAAAGTTAAAGGCCGCTTTTTAATTTT
Top_B	b	**GTTTGATC**CGTCGAGAGGGTTGATATATTTTTCAAATAT**TATCGTAA**
Top_B	b	**GATGGAGA**CTTTGCCCGAAGCGGATTGCATTTTTTTTGCAAAAGA
Top_B	b	**GCTTACTG**GTGGACTCCAACGTCATCCACTATTAAAGAAC**AAATGTTC**
Top_B	b	**TACCTGAG**GTGTTTTTATAAAACAGGAAAAAC
Top_B	b	**CGGTTGCT**GCTGCGCGTAACCACCACTACGCCAGAATCCTGAGAA**ATACTAGC**
Top_B	b	**GTATGTGC**TTAATGCGCGAAAATATCAAA**TATGACTT**
cyl		GGTGCCGTAAAGCACTAAATCGGAACACCCAAA
cyl		TTACTAGTGTGATAGCGCAGTCTCTGAA
cyl		ACGCTAATACAGAGTTATCAACAATAGA
cyl		AACAACGCTGTTTAGTATCATATGCGTTGAATCGCGTCCAGA
cyl		GGGCTAGCCACCAGCCGCCGCCAGCAATACATGTCTGACCGAACGCG
cyl		CTAAGAACGCGAGGCGTTTTAGCGAACAGATATCAATAAT
cyl		CGGCTGTAATATCCTTTTGTTTAACGTC
cyl		GACGGGGAAAGCCGGCGAACGTTTTTATAGTTGCTCTTACCA
cyl		TAAGTCCAAAATAGAAGTCAGAGGGTCTTATTAGC
cyl		AGAGCTTTCATTACTTGAAGCCTTTCCAGAGCCTA
cyl		CCTCAGAACCGCCACCCTCAGTAATTGAATACAAA
cyl		AGTACCGAGGCTGAGACTCCTCAAGAGAAGGATTA

cyl	AGCCAGAATGGAAAAATAAGGAACTATA
cyl	AGAGAATATAAAGTACCGACAAAAGAAAAATCTTTCCT
cyl	TATCATTGGCGCGTACTATGGGCTAAACCTACGTG
cyl	TTCTTACTTCATCTGCTTTTGACGGGGTCAGTGCCCGGGGTT
cyl	AGAAAACAGTATAGCCCGGAAACCCATGCCACAGA
cyl	AGAATAAATTAACTGAACACCCTGAACA
cyl	CCTGCCTTATTAAGCCACCCTCAGAACCGTTATAT
cyl	TCAATAGAATCGCAATCACCGTACTCAGGGGATAG
cyl	GGATTAGTTGAGTAAAGCGTCTTGACAGGAGGTTG
cyl	CAGCCTTCGAGCGTCTTAAATCAAGATT
cyl	TTTCATCTAAACCAAGTACCGCGCCTGT
cyl	TTGCTCAGTACCAGAGTTTTAATGATAC
cyl	AGGCAGGTTTACCGTTCCAGTACAGTGC
cyl	TCTGAGATGAGTGATTGAATTACCTTTT
cyl	ACATCCACGAGCTGAAAAGGT
cyl	AAAAATGTGAACAAGGTAAAGTAATTCTCATATTT
cyl	TGCGCTCACTGCCCTTTCTTTGTCCATCCGATTAA
cyl	ATACGTGGCACAGAACATCGCGCATCAC
cyl	ACCGAGCTCGAATTAGCTGATGCCGGAG
cyl	AAAACAGACAGTGCTTAAGCAAAATGGTCAATAACACAGTTG
cyl	GCTATATTTTCATTTGGGGCGATAAATC
cyl	ATGCGCCAGGGATTGTCTATCTGTTCCAGTTTGGA
cyl	ATACAGGATGAAAAATCTAAACATTAAA
cyl	GAGTTGCAGCAAGCGGTCCACTGAGTGTAGGGCGA
cyl	GTATTGGTTGTAGCAGCGGGATTGCTTTGACGAGCACGTATAACGTG
cyl	CCCTTATAAATCAAAAGAATAAACCATC
cyl	AAGAATTCTTTTGCGGGAGAAGCCTTTA
cyl	AAGCCTGTTTTGACCCGCCAGCCATTGCTCAGTGA
cyl	AATATTAGCTCAATCGTCTGAAAGCATA
cyl	AAGGCTACCCTAAACAATATTTTTGAAT
cyl	TCACCAGTGAGACGATTGCGTAAGTGTAAAGAGAA
cyl	TATTTACCTGAAAGAACGAACCACCAGCTGCCGGA
cyl	GCTACAGCCAAGAACGGGTATGTAGGAA
cyl	ACGCAAACTTGCTGGTAATATCCAGAACAGAGTCT
cyl	ATCTACAGCCTGAGGCAAGGATAAAAATAGCATTACTTGCTG
cyl	TCACCGCCTGGCCCTGAGAACAAGAG
cyl	ATAGAAAAAAAAAAGGCTCCATATATTCAAGAAAA
cyl	GGAAGGTAGAGCCAGCAGCAACAAGGCA
cyl	TGGCTTAGCGTTTTTCAGGTCTTTACCC
cyl	AATTACACCACGCAGGGTAGCAACGGATGAGCAAC
cyl	CGCAGAGACAGAAACAGACGACGATAAATCAGCAGCGAAAGA
cyl	AAGGGCGAAAAACCTTAGACAGAGTAAA
cyl	GGTCGCTGAGGCTTATCTCCAGGAACAA
cyl	GAGCCACGTCGTCTGGATTTTGCTAAAC
cyl	GGTCAGTTGGCAAATACAAACCTAAAGTATTGCTG
cyl	GGAACGATAACCGAAAAGGAGCCTTTAATTGTAT
cyl	AGTTTTGAGAAGCAAACGTTATTAATTTAATGGAAACGTAAA
cyl	ACGGTGTCTGGAAGGAAGTATACATTAT
cyl	ACTATCAACTGGATAGCGTCCCCATAAA
cyl	AATATAAGCCCGAAAGACTTCAAATATCGAGCTTA
cyl	ATGAATTTTCTGTATGTTCCAGAATATATG
cyl	TCAGATGATGGCAATTCATCAAACGTCACGCCTGAATTTCAT
cyl	TCAAAAAAATTCGAGCTTCAAGTCATTTTTGCGGA
cyl	ATAACCTAAGTTTTCACCCTCATTTTCAGAGGTTT
cyl	AACTTTCCGATCTATGCTTCTGTAAATC
cyl	TACCTTTAATTGCTATTCTGCATTTCGC
cyl	AGCGTAAAACAGTTTCAGCGGAGTGAGACAGCCCT
cyl	GCGAATTATGAAACCTTAGAATAGCATTTACCGTAACACTGAGTTTCGTCACCA
cyl	GAATTGAGGATTTATTTCATTCCATATACTGTTTA
cyl	TGACTATTATAGTCCCAGAGGGGGTAATAGTAAAATGTTTAGTAACCCT
cyl	CAGCATCCGTTTACTAAAGAATTATACTTCTGAATTAAAAGT
cyl	CCTGATTCATTTTGCGGAACACATTTGA
cyl	CATAGTTCAAGCCCAATAGGATAGGTGTAGACAAA
cyl	CAAAATTGTCGCTATTAATTAAGAAGAG
cyl	GATAATAAAGAAACCACCAGAGAAAACGAGAATGA
cyl	GAGGGTAGCTATTTAATACCGCGTAAGA
cyl	TTGAGAGTCGATGAACGGTAAGAGCCGGAATGGAT
cyl	TTCACGTTGAAAGCAGGGAGAAGATGATTCATT
cyl	GTTTGCCATCTTTTCCGGAACAGGCAG
cyl	ACTCCAACTTTAAACAGTTCAAGGAGCGTAGAGCCTTAGG
cyl	CGACGACAATAAACAACATGTTCAGCTTTTAACGCTCAACAGTA
cyl	AAATAAAAACGATTCATCCTAATTTACGAGCATTTTTCCAGTAATA
cyl	GTTTGGAATTGCGTAAGTTACAAAATCG
cyl	TTAATGGAAACATTTTTACATCGGGAGAAACAATA
cyl	GGCAACAGGCCACCGGAACGGACCCGCCGCGCTTA
cyl	CTTATCCGGTATTTTTTTGTAGAAACCAATAGAAGG
cyl	GGAATTATACAGTAACAGTACCTTTTTTTATTCCTGATTA
cyl	GGCTATTAGTCTTTTTGCTCATGGAAAT
cyl	CGTTAGTAATTTTAGAACCGCCACCCTCA
cyl	TCAATTACCTTTTATCAAAATTATTTGCGGGTTAGAACCTACCA

cyl		TAGATACGAACGAGTAGATTTAGTTTTATTAGAGAG
cyl		AACCAAAATAGCGAGAGGCTTTTTTTTGCGGGATCGTCACCC
cyl		GACTACCTTTTTAACCTCCTTTTGTACATAAATCA
cyl		TCAAGTTTTTTGGGGTCGATTTTAATCGGCAAAAT
cyl		GCGGTCAGTTTTTGGGACATTCTGGCCAAACGACCA
cyl		TAGTAATAATTTTTTCTTTCCTCGTTAGAATCAG
cyl		TCAGGTCATTGCTTTTCATATATTTTAAATGCAAT
cyl		GAGCCGCCATTTTCGCATTAGACGGGAGACATAAAA
cyl		TTGATATTCACAAACAAATAAATTTTTGGAACCAGA
	^FRET!	TTGATATTCAC/iCy3/AAACAAATAAATTTTTGGAACCAGA
cyl		GCCACCACATAATCAAAATCACCTTTTCCCAATCCA
cyl		GAATACCACATTCAACTAATGCATTTTAGCTTGATA
cyl		ATAAAGCCTCAGAGCATAAAGCTTTTTTTTGACCAT
cyl		ACCTACAGGGTGCCTAATGTTTTCTGAGAGTCTGGAGCAAAC
cyl		GTAATAAAATTTTCATCACTTGCCTGAGTCTTTGAT
cyl		AGCACTAACAATTTTATTAACACCGCCTGCAAGGTGAG
cyl		GCTGATTGCCCTTTTTAGTGAGCTAACTCACATTA
cyl		AGGAGTGTATTTTCCAGAACCACCACCAGACCCTCA
cyl		GGCTTAGGTTGGGCCACCCTCTTTTTTCTGAAACATGAAAGATTTCGG
inner		CCTAAAGGGAGCCCCCGATTTTGGCCCAAGGAGGC
inner		TTATCAAAATCATAGGTGTAAATGCTGATG
inner		TAAATTTAATGGTTTGAAATACAAATCCTGAAT
inner		AATACTTAGAAGAACAGTCACCAGAGATAGAACCC
inner		CAGTTGAACAACAACCATCGCTTTAACATTGCTTT
inner		TTCTGACATTGGCAGATTCACCTCAAAC
inner		ATGACCCTGTAATAAGCAAAACACGCTGTATCTAA
inner		AGGCATTTTCGAGTTTTATAAACACCGGAATCATAA
inner		TTGAGTATAGACTTTCAACAGTTGAAAG
inner		GAATACCAGATTTTCAGGTTTATATAAT
flap_struc		AGCACTATCACCGTTTTTAAGGGCGACATTCAAC
flap_struc		CAATGAAACCATTTTATTTGCCACTTGCG
flap_struc		ATTACGCAAAGGTGGAATAAGTGGTTTACCAGCGC
flap_struc		TTTCGGTCATAGCCTTCGATAGC
flap_struc		ATAAAAGAATTTTTCATGATTAAGACTCCTT
flap_struc		CGATTGACATTAAATTGGGAATTAGAGC
flap_struc		GAGCGCTAACTATCTTACTTTTCAGACGCTCAGAGCCGCCAC
flap_struc		TGTCACAATTTTTTAGAAAATACATACAT
flap_struc		CAGCAAAATCTTTTATTGACGGAAATTATTGGGAGGG
flap_struc		AAGGTAAATTTTTCAATAGAAAATTCATATTTATTT
flap_struc		CAAAGACAATTTTACGCAAAGACACCACGGCAACAT
flap_struc		AGAGGGAAAGATTTAACTGGCTCATTATACCAG
flap_struc		TAGTAAATTTTTTCCAAATCAACGTAACAACCGGAT
flap_struc		GGTCAATCATTTTATCATCGCCTGATAAATTGTGTCGAAACGAGGACCAACT
flap_struc		GCTTGCCCTGACGAGATCATTCAGT
flap_struc		AGGCGCAGACAAGAAAGCTGCAACACCAGAACGAG
flap_struc		CGGAGATTTGTTAACCAGACCGGAAGCAA
flap_struc		GAATAAGTTTTCATCAAGAGTAATCTTTAGGCTG
flap_struc		GCTGACCTTTTTTTAAGGGAACCGAACTGCGCAGAC
flap_struc		TAATGGACCGTTTTAGCCAGCTTTCATCA
flap_struc		AACGCCAGTGCTGCAAGGCGATTTTGAGAAAGAAATTAAAGAAGAT
flap_struc		GCACCGCTTCTGGGAAGGGCGATCGGTCAGACTGATGTTGATCGCTATTAC
flap_struc		ACGATGTACCGTCGACTCTAGAGGATCCCTTTTTTTAAGTTGGGT
flap_struc		GGGGGATGGGTTTTAAACCAGGCAAAGCGCCATTTTTATCGGCCTCAGG
flap_struc		TTAAATTAAATTTTACAAACGACATTAAATGTGA
flap_struc		GTTAAAATCTTCCTGTTGCAGGCGAAAATCCGGTTTGC
flap_struc		GCGGATTGGATAGGGACGACGACAGT
flap_lip	Pal	ACGAAAGAGGCAAAAGAATAGTACAA
flap_lip	Pal	TCGCCATTCAGGCTGCGCAACTGTTGGTGCCGGCCCAGTCTTGCATG
flap_lip	Chol	AAGATCGCACTCCATGCCAGTATAATC
flap_lip	Pal	TGACCCCTTTTAAACGGGTAAAATACGTAAAAC
flap_lip	Pal	CACCGACTTGAGCCATGGTGAATCGTAATCTCGGCAT
flap_lip	Pal	GTTAATAAAACGAACTAACTCA
flap_lip	Pal	ATCAGTCTCATGGGCGCATCGTTTTCGTCGGATCTCAT
flap_lip	Pal	CCTGCAGCCGGTTGTTGAGGGTCACGTTGGTGTAGCGTGGGATGTTAA
flap_lip	Chol	ACAGGTACTTTGAGGACTAAAACGAAGG
flap_lip	Chol	CAAAAACTTTTACGGCCAGTGCCAAGCACGACGTTGTAAAACG
	^FRET!	/5Cy5/CAAAAACTTTTACGGCCAGTGCCAAGCACGACGTTGTAAAACG
flap_lip	Chol	ATTCATTACTTTTGAACGGTGTGAAGTTTCCATT
flap_lip	Pal	CGAAGCCCTTTTTAAGAAAAATAACG
flap_lip	Chol	ACTCTAGCCGGAATCCGCGACCTGCTCTTTTTTTTCAGCGATTATAC
flap_lip	Chol	AGTATGTTAGCAAACGTCAACATCAGTCTGATAAGCTACCAGAAGGAAAC
flap_lip	Pal	CACTGACTTTTGGAACAACATTATT
flap_lip	Chol	TTTTTAATTTTAGGAAGATTGTATAAGCAAATAT
flap_lip	Chol	TGAGACAGACCTTGAAAGAGGACAGATTTTTCATGTTACTATCTT
flap_lip	Pal	ATAACCCGTTTTCAAGTAGCGGCCGGAAACGTCAC
flap_lip	Chol	CAAGAAAAGATAGCCGAACAAAGTTATTTTCAATAATAAGAG
flap_lip	Chol	TGAAATAGCAATAGTATCAGAGAG
flap_lip	Chol	TAGCGTCAGACTGTAGCGCACAAGAATTGAGTTAAGCC
flap_lip	Chol	GCGAGTAACAACCCAGACTGATGTTGACCAATAGGAACG
flap_lip	Pal+Lock	AGAAACATCGCCAGCTTTCCGTTTTTAACCGTGAGCCC

flap11_lip	Pal+Lock	**CCCAAAAGCAGCTG**GCCAGCTGGCGAAA
flap31_lip	Pal+Lock	**CCCAAAAGCAGCTG**CGAGGAAACGCAATAGTAAGCCAA
flap22_lip	Pal+Lock	**CCCAAAAGCAGCTG**TAATCATGTTGGGAAGAAAAATCTACTTTTTAATTTCAACTT
flap32_lip	Pal+Lock	**CCCAAAAGCAGCTG**TAGCAAGACAGAATCAAGTTTGCCTTTTTTCACCATTACCAT
flap12_lip	Pal+Lock	**CCCAAAAGCAGCTG**CCATCAAAAATAATTCGCGTCTGGCTCGCATTGTAA
flap21_lip	Pal+Lock	**CCCAAAAGCAGCTG**CAAGCGCGAAACAAACACTAATGC
cyl11	LockToe	AATCACCATCAA**CAGCTGCTTTTGGGATTCCGTTG**
cyl21	LockToe	CTAATAGATGAATTATCATCATTTCAAATGCAGGT**CAGCTGCTTTTGGGATTCCGTTG**
cyl12	LockToe	CCAACGCGCGGGGAGAGGCTGTTTGATGGTGGTTCCGA**CAGCTGCTTTTGGGATTCCGTTG**
cyl31	LockToe	CGTTAAATAAGATTTCATTAAAACCTATTA**CAGCTGCTTTTGGGATTCCGTTG**
cyl22	LockToe	CCGATAGGGTGAATTTCTTAAAC**CAGCTGCTTTTGGGATTCCGTTG**
cyl32	LockToe	AATAAGACAGCCATATTATTTAT**CAGCTGCTTTTGGGATTCCGTTG**
cyl_lip	Pal	AAATCGGTTGTACCAAAAACATTACAGTCA
cyl_lip	Chol	ATGGTCATAGCTTTTTTATGATATTCAACCGTTCTCGTAATC
cyl_lip	Pal	TATCGGCTTAACCGGCGCCAGGGTGGTTGCTTTCCAGTCGGG
cyl_lip	Chol	CCGACCGAAAAAGCCCAACATGTAATTTCGCCTCCATTGGCC
cyl_lip	Pal	ACAATTCCACACAACATACTCGTAAAACCGGGT
cyl_lip	Pal	TTTCAACTAATGTGTAGGTAAAGATTCAAAAGGGT
cyl_lip	Pal	TCAGGACTGTGAATTACCTTATGCGATTTTAATTCGGTTTATCAGCTTG
cyl_lip	Pal	ACGGATTGATGAATACGAGGCATAGTAA
cyl_lip	Chol	GTCATAAATATTCATTGAATCCCTTTTGATACATAACGCCAAAA
cyl_lip	Chol	GGAGGTTCGCGCCCAATAGCAAGCAAATCCTCCCGAGTTACA
cyl_lip	Pal	TCAATCATTTAATATTTTTAAACCTGTCGTGCCTATCCGCTC
cyl_lip	Pal	CACCAACCTAAATAATACTGCGGAATC
cyl_lip	Pal	AATATCTGTCAATAATTCCCACCTTTTGATAAGAGAGCG
cyl_lip	Chol	CTTTCGATTGCGCCGACAATGGATTTAG
cyl_lip	Pal	GAATACCCAAAATTTTTCCGTATAAACAGTTAATGCCC
cyl_lip	Chol	TTAATGAATCGGTTTTGTTTCCTGTGTGAAATTGTAGCTGCA
cyl_lip	Pal	GCCCGAGATAGGGTGCTGGTTTGCCCCA
Top_dye_Ha	Dye_handle	**TAACAGGATTAGCAGAGCGAGG**CTGGTAATAGCGGATAAGTGC
Top_dye_Ha	Dye_handle	**TAACAGGATTAGCAGAGCGAGG**TCAAAAAGATTAAGAGGAATGCTGTAGCTCA
Top_dye_Ha	Dye_handle	**TAACAGGATTAGCAGAGCGAGG**ACAAGCAAGCCGTGGCGAGAAAGGAAGGGAA
Top_dye_Ha	Dye_handle	**TAACAGGATTAGCAGAGCGAGG**ATTTTAGTTAATCAGTATAAAGCC
Top_dye_Ha	Dye_handle	**TAACAGGATTAGCAGAGCGAGG**CCCTCAATCAATATCTAACCTCACTGATAG
Top_dye_Ha	Dye_handle	**TAACAGGATTAGCAGAGCGAGG**GTGTTTTTATAAAACAGGAAAAAC
Top_dye_Ha	Dye_handle	**TAACAGGATTAGCAGAGCGAGG**ACTAATAGTAGTTTTTAGAACCCT
Top_dye_Ha	Dye_handle	**TAACAGGATTAGCAGAGCGAGG**GTGGACTCCAACGTCATCCACTATTAAAGAAC
Top_dye_Ha	Dye_handle	**TAACAGGATTAGCAGAGCGAGG**TTAATGCGCGAAAATATCAAA
Top_dye_Ha	Dye_handle	**TAACAGGATTAGCAGAGCGAGG**TCACCGCCTGGCCCTGAGAACAAGAG
Inner(2)	Immob_handle	CCTAAAGGGAGCCCCCGATTTTGGCCCAAGGAGGC**CATGCGAGGCTACACT**
Inner(2)	Immob_handle	TTATCAAAATCATAGGTGTAAATGCTGAT**CATGCGAGGCTACACT**
Inner(2)	Immob_handle	TAAATTTAATGGTTTGAAATACAAATCCTGAAT**CATGCGAGGCTACACT**
Inner(2)	Immob_handle	AATACTTAGAAGAACAGTCACCAGAGATAGAACCC**CATGCGAGGCTACACT**
Inner(2)	Immob_handle	CAGTTGAACAACAACCATCGCTTTAACATTGCTTT**CATGCGAGGCTACACT**
Inner(2)	Immob_handle	TTCTGACATTGGCAGATTCACCTCAAAC**CATGCGAGGCTACACT**
Inner(2)	Immob_handle	ATGACCCTGTAATAAGCAAAACACGCTGTATCTAA**CATGCGAGGCTACACT**
Inner(2)	Immob_handle	AGGCATTTTCGAGTTTTATAAACACCGGAATCATAA**CATGCGAGGCTACACT**
Inner(2)	Immob_handle	TTGAGAGTCGATGAACGGTAAGAGCCGGAATGGAT**CATGCGAGGCTACACT**
Inner(2)	Immob_handle	GAATACCAGATTTTCAGGTTTATATAAT**CATGCGAGGCTACACT**

Bio_plug capture oligo	AGCTCACTCAGGACGTTCAATTACCTTTTATCAAAATTATTTGCGGGTTAGAACCTACCA<br>CGACGACAATAAACAACATGTTCAGCTTTTAACGCTCAACAGTAAGCTCACTCAGGACGT
3'bio	TACCTGTCGACGTCCTGAGTGAGCT
Bio unplug oligo	AGCTCACTCTGGACGTCTACAGGTA
Dye oligo	/DYE/CCTCGCTCTGCTAATCCTGTTA
Key oligo	CAACGGAATCCCAAAAGCAGCTG
Scramble key oligo	TAGCTTATCAGACTGATGTTGA
PEG plug oligo	/5AmMO/AGTGTAGCCTCGCATG**AGCCC**
Unplugging oligo	**GGGCT**CATGCGAGGCTACACT

Supplementary References

1. Douglas, S. M. *et al.* Rapid prototyping of 3D DNA-origami shapes with caDNAno. *Nucl. Acids Res.* **37**, 5001–5006 (2009).

2. Langecker, M. *et al.* Synthetic Lipid Membrane Channels Formed by Designed DNA Nanostructures. *Science* **338**, 932–936 (2012).

3. Göpfrich, K. *et al.* Large-Conductance Transmembrane Porin Made from DNA Origami. *ACS Nano* **10**, 8207–8214 (2016).

4. Wohlert, J., den Otter, W. K., Edholm, O. & Briels, W. J. Free energy of a trans-membrane pore calculated from atomistic molecular dynamics simulations. *The Journal of Chemical Physics* **124**, 154905 (2006).

5. Krishnan, S. *et al.* Molecular transport through large-diameter DNA nanopores. *Nat Commun* **7**, 12787 (2016).

6. Chiba, M., Miyazaki, M. & Ishiwata, S. Quantitative Analysis of the Lamellarity of Giant Liposomes Prepared by the Inverted Emulsion Method. *Biophysical Journal* **107**, 346–354 (2014).

7. Hatzakis, N. S. *et al.* How curved membranes recruit amphipathic helices and protein anchoring motifs. *Nat Chem Biol* **5**, 835–841 (2009).

8. Mortensen, K. I., Tassone, C., Ehrlich, N., Andresen, T. L. & Flyvbjerg, H. How To Characterize Individual Nanosize Liposomes with Simple Self-Calibrating Fluorescence Microscopy. *Nano Lett.* **18**, 2844–2851 (2018).

9. Song, Langzhou & Hobaugh, Michael R. & Shustak, Christopher & Cheley, Stephen & Bayley, Hagan & Gouaux, J. Eric, Structure of Staphylococcal α-Hemolysin, a Heptameric Transmembrane Pore, Science 274, 1859-1865 (1996)

10. Tonnesen, Asger Christensen, Sune M. & Tkach, Vadym & Stamou, Dimitrios, Geometrical Membrane Curvature as an Allosteric Regulator of Membrane Protein Structure and Function, Biophysical Journal 106, 201 – 209 (2014)
