# Supplementary material for: A large size-selective DNA nanopore with sensing applications
Source: Nat Commun. 2019 Dec 11;10:5655. doi: 10.1038/s41467-019-13284-1 (PMC6906287; doi:10.1038/s41467-019-13284-1)
Supplement: Supplementary file 4 — Reporting Summary [file 41467_2019_13284_MOESM4_ESM.pdf]

nature research

Corresponding author(s): Jørgen Kjems (jk@mbg.au.dk)
Nikos Hatzakis (hatzakis@nano.ku.dk

Last updated by author(s): Oct 8, 2019

Reporting Summary

Nature Research wishes to improve the reproducibility of the work that we publish. This form provides structure for consistency and transparency in reporting. For further information on Nature Research policies, seeAuthors & Referees and theEditorial Policy Checklist .

Statistics

For all statistical analyses, confirm that the following items are present in the figure legend, table legend, main text, or Methods section.

n/a	Confirmed	
☐	☒	The exact sample size (*n*) for each experimental group/condition, given as a discrete number and unit of measurement
☐	☒	A statement on whether measurements were taken from distinct samples or whether the same sample was measured repeatedly
☐	☒	The statistical test(s) used AND whether they are one- or two-sided<br>*Only common tests should be described solely by name; describe more complex techniques in the Methods section.*
☒	☐	A description of all covariates tested
☒	☐	A description of any assumptions or corrections, such as tests of normality and adjustment for multiple comparisons
☐	☒	A full description of the statistical parameters including central tendency (e.g. means) or other basic estimates (e.g. regression coefficient) AND variation (e.g. standard deviation) or associated estimates of uncertainty (e.g. confidence intervals)
☐	☒	For null hypothesis testing, the test statistic (e.g. *F*, *t*, *r*) with confidence intervals, effect sizes, degrees of freedom and *P* value noted<br>*Give P values as exact values whenever suitable.*
☒	☐	For Bayesian analysis, information on the choice of priors and Markov chain Monte Carlo settings
☒	☐	For hierarchical and complex designs, identification of the appropriate level for tests and full reporting of outcomes
☐	☒	Estimates of effect sizes (e.g. Cohen's *d*, Pearson's *r*), indicating how they were calculated

Our web collection on statistics for biologists contains articles on many of the points above.

Software and code

Policy information about availability of computer code

Data collection: Origami Design: caDNAno2. TEM collection: EMmenu 2.0 and EPU software (cryoEM). Confocal measurements: Leica LASX black software. Gel imaging: Amersham Typhoon scanner software 2.0. 'DLS: DLS Zetasizer software 7.12, TIRF operation: Olympus cellSens. HPLC: Agilent Chemstation. FRET measurements: FluoroMax software. Flow cytometer: see below.

Data analysis: TEM averaging: Scipion. Confocal: Leica LASX black and Image G. Gel imaging: Imagequant TL 8.2, DLS: DLS zetasizer software 7.12 (export to Excel). TIRF: Tracking and localization: Code available on Hatzakis group homepage (https://hatzakisgroup.github.io/). HPLC: agilent chemstation (export to Excel). FRET measurements: Fluoromax software (export to Excel).

For manuscripts utilizing custom algorithms or software that are central to the research but not yet described in published literature, software must be made available to editors/reviewers. We strongly encourage code deposition in a community repository (e.g. GitHub). See the Nature Research guidelines for submitting code & software for further information.

Data

Policy information about availability of data

All manuscripts must include a data availability statement. This statement should provide the following information, where applicable:

- Accession codes, unique identifiers, or web links for publicly available datasets
- A list of figures that have associated raw data
- A description of any restrictions on data availability

The authors declare that the data supporting the findings of this study are available within the paper and its supplementary infor-mation files.

Field-specific reporting

Please select the one below that is the best fit for your research. If you are not sure, read the appropriate sections before making your selection.

☒ Life sciences ☐ Behavioural & social sciences ☐ Ecological, evolutionary & environmental sciences

For a reference copy of the document with all sections, see nature.com/documents/nr-reporting-summary-flat.pdf

Life sciences study design

All studies must disclose on these points even when the disclosure is negative.

Sample size	No sample size calculation
Data exclusions	No data excluded relevant to the manuscript
Replication	All experiment was replicated several times to achieve enough statistics. Structure annealings done at multiple universities by instructed people.
Randomization	Not relevant
Blinding	Blinding in most cases not done as the prepared samples was analyzed by the same person.

Reporting for specific materials, systems and methods

We require information from authors about some types of materials, experimental systems and methods used in many studies. Here, indicate whether each material, system or method listed is relevant to your study. If you are not sure if a list item applies to your research, read the appropriate section before selecting a response.

Materials & experimental systems

n/a	Involved in the study
☒	☐ Antibodies
☒	☐ Eukaryotic cell lines
☒	☐ Palaeontology
☒	☐ Animals and other organisms
☒	☐ Human research participants
☒	☐ Clinical data

Methods

n/a	Involved in the study
☒	☐ ChIP-seq
☐	☒ Flow cytometry
☒	☐ MRI-based neuroimaging

Flow Cytometry

Plots

Confirm that:

☒ The axis labels state the marker and fluorochrome used (e.g. CD4-FITC).

☒ The axis scales are clearly visible. Include numbers along axes only for bottom left plot of group (a 'group' is an analysis of identical markers).

☒ All plots are contour plots with outliers or pseudocolor plots.

☒ A numerical value for number of cells or percentage (with statistics) is provided.

Methodology

Sample preparation	not cells - GUVs prepared by Inverted emulsion assay as described in methods
Instrument	Beckman Coulter Galios
Software	Kaluza Flow Cytometry Software
Cell population abundance	Not cells
Gating strategy	No gating - full population.

☐ Tick this box to confirm that a figure exemplifying the gating strategy is provided in the Supplementary Information.
