# Supplementary material for: A large size-selective DNA nanopore with sensing applications
Source: Nat Commun. 2019 Dec 11;10:5655. doi: 10.1038/s41467-019-13284-1 (PMC6906287; doi:10.1038/s41467-019-13284-1)
Supplement: Supplementary file 5 — Description of Additional Supplementary Files [file 41467_2019_13284_MOESM5_ESM.pdf]

Title: Supplementary Movie 1

Description: Video displaying direct observation of single liposomes for over 8 hours and correction of inherent stage drift (see additionally supplementary Fig. S19). Left: Full field of view for drift corrected observation. Middle: Zoom in on area containing multiple liposomes, displaying correction for stage drift using the software as compared to the non-corrected zoom (right).
